# Reconstruction and signal propagation analysis of the Syk signaling network in breast cancer cells

**DOI:** 10.1371/journal.pcbi.1005432

**Published:** 2017-03-17

**Authors:** Aurélien Naldi, Romain M. Larive, Urszula Czerwinska, Serge Urbach, Philippe Montcourrier, Christian Roy, Jérôme Solassol, Gilles Freiss, Peter J. Coopman, Ovidiu Radulescu

**Affiliations:** 1 DIMNP, Dynamique des Interactions Membranaires Normales et Pathologiques, CNRS, UMR5235, Montpellier, France; 2 Université de Montpellier, Montpellier, France; 3 IRCM, Institut de Recherche en Cancérologie de Montpellier, Montpellier, France; 4 INSERM, U1194, Montpellier, France; 5 Institut régional du Cancer de Montpellier, Montpellier, France; 6 Plateforme de Protéomique Fonctionnelle (FPP), Institut de Génomique Fonctionnelle, Montpellier, France; 7 Institut de Génomique Fonctionnelle (IGF), CNRS UMR5203, Centre National de la Recherche Scientifique (CNRS), Montpellier, France; 8 Inserm, U1191, Montpellier, France; 9 CHU Montpellier, Arnaud de Villeneuve Hospital, Department of Pathology, Montpellier, France; Duke University, UNITED STATES

## Abstract

The ability to build in-depth cell signaling networks from vast experimental data is a key objective of computational biology. The spleen tyrosine kinase (Syk) protein, a well-characterized key player in immune cell signaling, was surprisingly first shown by our group to exhibit an onco-suppressive function in mammary epithelial cells and corroborated by many other studies, but the molecular mechanisms of this function remain largely unsolved. Based on existing proteomic data, we report here the generation of an interaction-based network of signaling pathways controlled by Syk in breast cancer cells. Pathway enrichment of the Syk targets previously identified by quantitative phospho-proteomics indicated that Syk is engaged in cell adhesion, motility, growth and death. Using the components and interactions of these pathways, we bootstrapped the reconstruction of a comprehensive network covering Syk signaling in breast cancer cells. To generate *in silico* hypotheses on Syk signaling propagation, we developed a method allowing to rank paths between Syk and its targets. We first annotated the network according to experimental datasets. We then combined shortest path computation with random walk processes to estimate the importance of individual interactions and selected biologically relevant pathways in the network. Molecular and cell biology experiments allowed to distinguish candidate mechanisms that underlie the impact of Syk on the regulation of cortactin and ezrin, both involved in actin-mediated cell adhesion and motility. The Syk network was further completed with the results of our biological validation experiments. The resulting Syk signaling sub-networks can be explored via an online visualization platform.

## Author summary

The complex nature of cancer hampers traditional biological approaches to unravel its molecular mechanisms and develop targeted drug therapies. Cancer affects a number of “hallmark” cellular processes controlled by multiple signaling pathways. Our goal is to identify the pathways that negatively affect tumor development and progression. We established that the Syk protein tyrosine kinase exhibits a tumor-suppressive function in breast cancer. Large scale global biochemical analyses allowed to identify Syk targets in cancer cells, but their mechanisms and interrelationships remain unknown. Our main goal was to pinpoint a limited number of biologically realistic molecular “paths” from Syk to its effectors. We therefore developed a new methodology combining graph theoretical methods allowing to reveal the shortest “paths” between “nodes” in a graph including an approach that investigates also longer “paths”. Applied to the Syk network, this method allowed us to propose and validate new signaling axes relating Syk to major effectors of the cell adhesion and mobility that are crucial cancer hallmarks.

## Introduction

Tyrosine phosphorylation of proteins acts as an efficient switch allowing to control key signaling pathways involved in cell proliferation, apoptosis, migration, and invasion, and is thus involved in oncogenesis. Understanding the functioning of such complex pathways is crucial for both fundamental research and clinical applications and relies on the ability to build in-depth network models from extensive global experimental data [1–7].

The non-receptor spleen tyrosine kinase Syk has for a long time been considered as a hematopoietic cell-specific signaling molecule. In these cells, Syk is involved in coupling activated immunoreceptors to downstream signaling events affecting cell proliferation, differentiation and survival [8]. We and others have discovered that Syk is also present in non-hematopoietic cells [9–12]. More precisely, its expression was found in mammary epithelial cells and low-tumorigenic breast cancer cell lines, whereas invasive and metastatic breast cancer cells lacked Syk expression [11]. In patient samples, Syk expression exhibits a gradual loss during breast cancer progression and the low Syk levels are correlated with an increased risk of metastasis [13,14]. In hematopoietic cells, Syk functions as an essential component of the signaling machinery of multiple immune receptors and adapter proteins that are, however, not expressed in non-hematopoietic cells. Unveiling the Syk signaling pathways and tumor suppressor mechanisms is a public health issue as pharmacological Syk inhibitors are being used in clinical trials for treating auto-immune diseases [15,16].

We and other groups performed quantitative phospho-proteomic studies, based on differential Syk expression or activity, in order to identify novel Syk signaling effectors in breast cancer cells [17–19]. These approaches, however, allowed only to establish a comprehensive list of direct and indirect Syk targets. In this study, we use the data produced in these investigations to reconstruct a Syk-based signaling network and to identify the intermediary pathways via which the signal propagates from Syk to its effectors in this network.

Phospho-proteomic studies provide data sets of phosphorylated proteins with a significant “fold change” in differential experiments. Comparably to the gene data sets in transcriptomic studies, these protein targets can be used to build networks by using comprehensive interaction databases (hypergeometric test [20]; GSEA [21]; DAVID [22]; enrichment maps [23]). On the one hand, the set of identified targets is incomplete and intermediate variables and interactions are needed to describe systems-level functioning. On the other hand, the set may be too substantial and contain spurious or inessential components. Consequently, in order to obtain comprehensive networks that expose new essential constituents, network reconstruction procedures should contain both enrichment and pruning steps.

Network pruning can be performed by sub-network extraction [7,24–27]. Three main approaches based on generic graph analysis methods have been used to extract sub-networks associated to a subset of its nodes: the classical shortest path [27–29], Steiner trees [7,25,26] and random walk processes [30]. Module identification using expression data to score sub-networks [31] is a closely-related method but its aim is to identify a number of significant small sub-networks rather than produce a simplified connected network.

Here we focus on the identification of signaling pathways from a single source to a collection of targets identified in phospho-proteomic studies. In this context, the classical shortest paths approach can be inaccurate and does not take into account alternative paths, while the k-shortest paths extension generates a collection of ranked alternative paths, but relies on well-separated weights between arcs to be effective [27]. Steiner trees enable the identification of the smallest set of edges allowing to connect a set of nodes: it may lead to longer individual paths but will reduce the number of interactions when considering the dataset as a whole, with the drawback of further reducing the number of alternative paths [25]. Suboptimal solutions of the Steiner tree problem were computed for a lymphoma network [32] but the functional significance of disparate solutions was not appraised. Finally, random walk processes have been used to estimate the probability of reaching network nodes by observing information flow propagation [30,33] The random walk approach can prioritize some proteins [30,34] but does not usually aim to identify paths from the source(s) to the target(s). Additionally, it can be inaccurate and fail to render specific features of the signaling pathways because it assumes that the flow of information through the network satisfies linear equations (Laplace equation on a graph) with transitions that are mainly guided by topology. Other turn-key tools have been developed, such as Ingenuity Pathways Analysis (IPA) [35]. IPA mainly involves gene-regulatory networks and request the signs of the regulations in order to score interactions and paths. The latter information is rarely available in relation with protein modification/interaction and phospho-proteomics data. Furthermore, IPA is a proprietary software with a private database. Despite their limitations, each of these methods provides valuable information and it is useful to combine several approaches with *ad-hoc* adjustments when analyzing networks in relation with specific data.

In this paper we assembled the interaction network of signaling pathways controlled by Syk in breast cancer cells, exploiting existing phospho-proteomic studies. To reconstruct and analyze the signaling network, we propose a novel methodology that combines the shortest paths methods with random walk processes. We defined interaction weights based on functional annotations and experimental datasets. Mainly, we pinpointed phosphorylation-based interactions leading directly to targets whose changes of phosphorylation are significant and interactions whose sources were identified targets. These weights define transition probabilities of a Markovian random walk on the network, which are further refined by replacing them with probability currents of the stationary Markov process. Thus, the random walk is not used for pruning directly, like in other implementations of this technique[30,33], but applied for weight re-evaluation. To produce a list of biologically relevant paths relating Syk to its direct and indirect targets, we then searched for near-shortest paths in the resulting network with refined weights. Our method combines the advantages of weighted shortest path methods that take into account the functional importance of the interactions with those of the random walk methods that propagate the information on the network and smoothen large weight differences that could incidentally occur. The increase in specificity obtained by combining several sub-network extraction methods has also been exploited for analyzing metabolic networks [36]. By network pruning, relatively dense networks are downscaled to a few biologically significant alternative paths from Syk to its targets. This helps to generate hypotheses about Syk signal propagation that, however, need to be experimentally validated.

We substantiated our *in silico* hypotheses with molecular and cell biology experiments, and identified two candidate mechanisms that support the impact of Syk on the regulation of cortactin and ezrin, two proteins involved in actin-based cell adhesion and motility. As a result of our biological validations, we propose a new Syk-Src-cortactin signaling axis, and a direct ezrin regulation by Syk phosphorylation. The Syk network was further corroborated with biological validation experiments and exploited to generate the sub-networks of the paths from Syk to its targets involved in (**i**) cell adhesion and motility, (**ii**) cell growth and death, (**iii**) immunity and inflammation and (**iv**) cell differentiation.

The proposed sub-network extraction method was applied to connect Syk, a source in the network, to its direct and indirect targets. The same method can be applied in numerous other studies to connect several sources amongst themselves and to their targets. This method reveals important targets and interactions, allows to generate hypotheses and test new interactions. The simplified network resulting from this method provides insights into biological processes controlled by Syk and provides a challenging access to more mechanistic modeling approaches.

## Results

Starting with the set of Syk-dependent targets identified by phosphoproteomics, we identified a network explaining the propagation of the signal from Syk to its targets. In order to generate this network we interrogated comprehensive pathways databases, extracted significantly enriched pathways and consistently merged them by avoiding duplicate interactions and nodes. The result of this assembly was a connected but very large network, too difficult to analyze and containing many unessential interactions. Here, we propose several methods allowing to prune this network and to extract significant paths from it. Several paths connecting Syk to functionally important targets were biologically validated.

### Selection of Syk targets in breast cancer cells

To bootstrap the reconstruction of a comprehensive network of Syk downstream signaling in breast cancer cells, we analyzed tyrosine phospho-proteomic data acquired in two independent mass spectrometric studies using breast cancer cell lines with modified Syk catalytic activity or protein expression. On the one hand, we selected from our own study the proteins lost or gained in tyrosine-phosphorylated protein complexes of the Syk-positive MCF7 cells treated with a pharmacological Syk inhibitor (further referenced as the MCF7 dataset) [19]. On the other hand, we identified the proteins with modified tyrosine phosphorylation after exogenous Syk expression in the Syk-negative MDA-MB-231 cells (further referenced as the MDA231 dataset) [17]. Post-treatment procedures of the original phospho-proteomic data are detailed in the Materials and Methods section. From these two studies; we respectively selected 265 and 487 proteins as Syk targets (S1 and S2 Tables). Only 64 proteins were found in common, reflecting the complementarity of the two original phospho-proteomic studies. Indeed, the phospho-tyrosine enrichment prior to mass spectrometry as well as the experimental cell models used in those studies are different (see the Materials and methods section). We analyzed the two datasets separately to evaluate whether they point to distinct or similar cell signaling pathways.

### Identification of overrepresented pathways

We searched for enriched pathways in the lists of Syk targets, using pathways from the KEGG database [37]. We selected pathways which contain at least one of the proteins from the target lists and used a Fisher exact test to assess their enrichment. As we are only interested in overrepresented pathways, we removed the underrepresented ones: i.e; the ones for which the ratio “number of proteins from the target list per total number of proteins” is lower than the same ratio in the background list.

Within the two rather poorly overlapping datasets, we found among the most enriched KEGG pathways those related to cell-cell and cell-substrate adhesions, actin cytoskeleton regulation and apoptosis (S3 and S4 Tables). This observation is consistent with the reported role of Syk on cell adhesion, motility, proliferation and death in breast cancer cells [19,38–43]. Furthermore, the similarity in pathway enrichment, despite the limited overlap between the two datasets, indicates their relationship. We merged the original datasets and present here the results obtained for the network reconstruction.

### Network reconstruction

**Fig 1:**
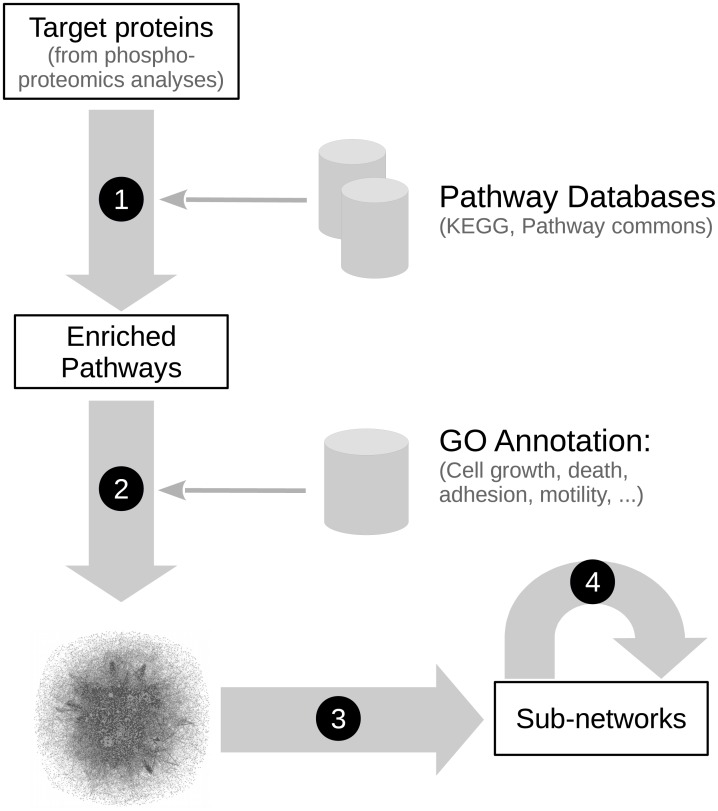
Workflow of the network reconstruction and signal propagation analysis. The combination of simple steps unravels candidate mechanisms based on a list of proteins identified by (phospho-) proteomic experiments. (1) Identification of enriched pathways in the target list. (2) Building of a large network integrating interactions from the selected pathways. Proteins are associated with manually selected keywords based on their GO annotation. These annotations reflect functional categories associated to the enriched pathways (cell adhesion & motility, cell growth & death, cell differentiation and immunity & inflammation) and highlight phospho-tyrosine modifiers (tyrosine kinases and phosphatases), which are important to decipher this specific dataset. (3) Extraction from this large network of sub-networks allowing to propose candidate mechanisms via the action of Syk on its effectors, by a combination of weighted shortest paths and random walk methods. (4) Biological validation of candidate mechanisms. The individual steps are detailed in the Materials and Methods section.

We assembled and explored a prior-knowledge interaction network to extract candidate mechanisms underlying the datasets. While such networks are often assembled using complete pathway or interaction databases, here we focused on previously identified enriched pathways. Using pathways rather than individual interactions will enable the extension of sub-networks with relevant interactions in their neighborhood for further analysis. By restricting our full network to a subset of pathways, some coverage may be lost but it allows to reduce the amount of irrelevant interactions and to better assess the relevance of the identified pathways. Hereafter we show that the enriched pathway can be used to identify candidate mechanisms. We also extended our search by using the Pathway Commons database [44]. KEGG provides a set of well-established pathways, while Pathway Commons allows a higher coverage by integrating pathways from multiple sources (notably Reactome, Panther, and PID). To select the most relevant pathways guaranteeing to cover most identified targets, we filtered the pathways based on their enrichment p-value and selected those presenting a p-value lower than 0.1 (in this step we aim to be as complete as possible, selecting 83 pathways from KEGG and 419 from Pathway Commons). Furthermore, we included the pathways containing Syk targets not covered by significantly over-represented pathways from the same database. This allowed to integrate 41 additional pathways from KEGG and 9 from Pathway Commons (S5 and S6 Tables). In this merged network, each protein and interaction keeps track of the list of pathways in which it is involved. We also used the GO annotation to identify the proteins involved in processes in which Syk is implicated (cell adhesion and motility, cell growth and death, immunity and inflammation, cell differentiation). The resulting oriented and partly signed network comprises 6438 proteins and 62322 interactions, from 552 pathways (124 from KEGG, 428 from Pathway Commons), covering 350 of the 687 identified targets (75 are only found in KEGG, 125 in Pathway Commons and 150 in both). Among these 350 targets, 245 are reachable from Syk (steps 1–2 in Fig 1). This network contains 979 interactions between two targets identified in the datasets. A closer scrutiny of these interactions further highlights connections between the two datasets: 146 and 260 interactions are associating targets specific to the MCF7 and MDA231 datasets respectively, 253 involve at least one target shared by the two datasets and 320 connect targets specific to different datasets.

### Sub-networks extraction by shortest paths analysis

The paths connecting Syk to the identified targets in the reconstructed network describe possible mechanisms for its signal propagation. As a massive amount of alternative paths exist, it is crucial to identify the appropriate candidates amongst the mass by extracting the sub-networks that exhibit selected connections between Syk and a specific target (step 3 in Fig 1; S1 Fig).

We focused on the signal propagation from Syk to its targets involved in cell adhesion and motility, and in particular on cortactin and ezrin, two proteins that are differentially phosphorylated in a Syk-dependent manner and that functionally link the plasma membrane to the actin cytoskeleton [45,46]. We first considered a parsimonious approach to find the paths implicating the fewest nodes, and searched for these shortest paths using the classical Dijkstra algorithm [47].

**Fig 2:**
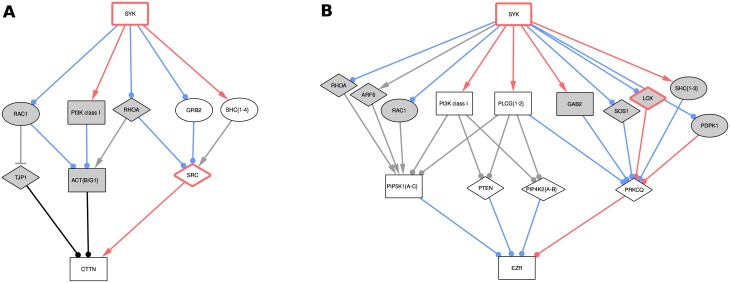
Proposed sub-networks for the effect of Syk on cortactin (A) and ezrin (B) identified by shortest paths analysis. The grey nodes are only involved in the unweighted shortest paths, while the white ones are also involved in the weighted ones. The red borders denote phospho-tyrosine modifiers (tyrosine kinases and phosphatases). Note that some related proteins have been grouped in single nodes for clarity (denoted by curly brackets at the end of the name, and “PI3K class I”). Interactions annotated as “phosphorylation” (not particularly on tyrosine) appear in red, while “protein modifications” in general appear in blue. Line ends indicate the sign of the interaction: arrow for positive, T for negative, and circle for unknown. Finally, node shapes denote identified proteins: squares have identified differentially phosphorylated peptides (MDA231 dataset), circles correspond to differentially enriched proteins (MCF7 dataset), and diamonds are not part of the datasets.

We observed that the shortest paths between Syk and cortactin contain two intermediates, with three alternative proteins directly upstream of cortactin (Fig 2A). Src is the only phospho-tyrosine modifier (tyrosine kinases and phosphatases) amongst them, suggesting that the paths involving Src are more credible to explain the change in cortactin phosphorylation.

The shortest paths network linking Syk to ezrin included more proteins and contained also two intermediate nodes (Fig 2B). These paths were related with the classical regulation of ezrin by the membrane lipid PIP2 or its phosphorylation on serine/threonine residue(s) [48,49]. None of the nodes directly upstream of ezrin were phospho-tyrosine modifiers that could explain the effect of Syk on ezrin tyrosine phosphorylation. The sub-networks obtained for cortactin and ezrin illustrate the need for careful analysis of phospho-tyrosine modifiers to explain the phosphorylation-based modifications noticed in the MDA231 dataset.

### Weighted shortest-path analysis

To take into account the importance of phospho-tyrosine modifiers, we extended the GO annotation of network nodes with the corresponding terms (S7 Table) and modulated the length of the path using distances attached to the interactions: a path involving more steps associated to small distances can be selected over a short path with longer steps. We assigned distances giving the priority to paths linking a phospho-tyrosine modifier upstream of proteins experimentally identified as differentially phosphorylated. We also favored the inclusion of other experimentally identified proteins by reducing the distances of their outgoing interactions.

The introduction of these distances led to more realistic suggestions for the cortactin sub-network, in which Src is the only protein directly upstream of cortactin (paths via white nodes in Fig 2A). As shown in the dedicated Results subsection, biological validation confirmed the role of Src in mediating Syk signal propagation to cortactin. However, it did not enable the identification of significantly better suggestions for ezrin, suggesting a “missing link” (paths via white nodes in Fig 2B). To resolve this inconsistency, we decided to evaluate the possibility of a direct interaction between Syk and ezrin. These results are described below and prompted us to propose that Syk directly phosphorylates ezrin.

Taking into account our biological validation, we added the new protein interaction from Syk to ezrin to the Syk network. We also extended the list of Syk direct substrates by integrating the results of a third dataset that identified the peptides phosphorylated on tyrosine by Syk after an *in vitro* kinase reaction [18] (S2 Table; for details, see Materials and methods). Finally, we added the direct interactions between Syk, E-cadherin and alpha-catenin, both members of the same cell-cell adhesion complex that we previously identified as direct Syk substrates [19] (step 4 in Fig 1).

### Path ranking by random walk refinement and overflow

The sub-networks obtained with the weighted shortest-paths analysis still contain several equivalent paths which we would like to classify further by integrating a parameter based on the network topology. A random walk process provides a “reachability” score for each protein. By taking into account both a mix of network topology and the weighted interactions, this score highlights key proteins that are involved in multiple interesting and plausible candidate paths. To integrate this parameter into our analysis method, we used these scores to refine the distances associated to their outgoing edges, allowing the shortest-path approach to also include such key proteins. The weight refinement algorithm is fully described in the Methods section.

**Fig 3:**
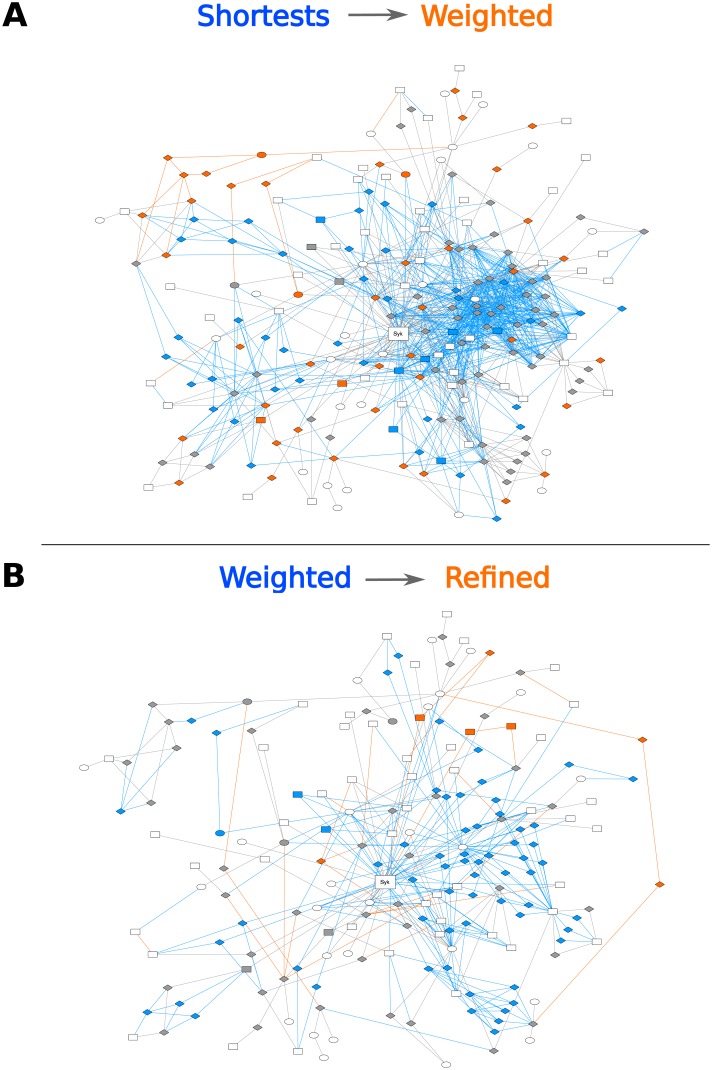
Successive refinements of the shortest-paths sub-networks associated to cell adhesion and motility. Evolution of the sub-network for the effect of Syk on proteins associated to cell adhesion and motility, starting with unweighted shortest paths, after the integration of weights (A), and by refining them using random walks (B). White nodes correspond to target proteins, grey nodes are intermediate proteins preserved by the refinement. The other colors denote nodes and edges that are affected by the refinement: blue elements are lost, while orange ones are introduced. The shape of nodes symbolizes the identification of the corresponding proteins in the original dataset, as in Fig 2. The ezrin and the cortactin sub-networks illustrated in Fig 2 are included in the adhesion and motility network.

This methodology was applied to refine analysis of the Syk signal propagation to its targets involved in cell adhesion and motility. The size of this sub-network (e.g. number of nodes and edges) decreased after modulation of the length of the paths and even more after random walk refinement (Fig 3). This ranking property was appropriate to highlight the most likely paths regarding our biological and topological criteria. These observations were confirmed by the sub-networks linking Syk to its targets involved in Syk-related cell processes (S2–S4 Figs)

**Fig 4:**
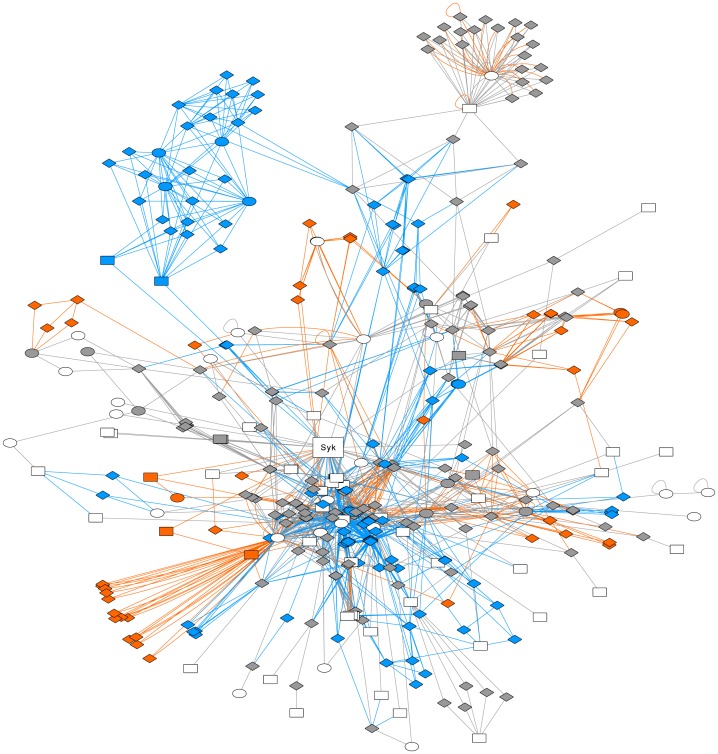
Extension of the weighted shortest-paths sub-networks. Evolution of the sub-network for the effect of Syk on proteins associated to cell adhesion and motility using weighted shortest paths, and after refinement with random walks, both allowing a 20% overflow. White nodes correspond to target proteins (which are all involved in cell adhesion or motility), grey nodes are intermediate proteins selected with the two approaches. The other colors denote nodes and edges that are specifically selected in one of the two methods: blue for weighted shortest paths, orange with random walk. The shape of nodes symbolizes the identification of the corresponding proteins in the original dataset, as in Fig 2. Although the new paths introduced by the overflow lie outside the cortactin and ezrin sub-networks, this methodological figure is useful for illustrating the flexibility of our approach.

Nevertheless, we considered this method as too stringent and searched for near-shortest paths instead of strict shortest paths in order to generate a set of alternatives, selecting all paths for which the total distance is up to 20% higher than that of the shortest path. Using this setting for the analysis of the signal propagation from Syk to its targets involved in cell adhesion and motility, we retrieved a sub-network as large as the one obtained without random walk refinement. Moreover, the sets of interactions linking Syk to its targets involved in Syk-related cell processes were slightly distinct as compared to the sub-networks obtained after weighted shortest paths analysis, and after refinement with random walks, both allowing a 20% overflow (Fig 4 and S5–S7 Figs). This suggests that introducing the “reachability” parameter not only ranks the alternatives, but also selects novel elements that allow to generate hypotheses on Syk signal propagation.

Taken together, the purpose of Figs 3 and 4 is to illustrate the flexibility of our method. These two figures tell us that (i) taking into account the molecular biology parameters by attaching distances to interactions for shortest-paths analysis and (ii) taking into account the network topology by the random walk refinement, leads not only to shortest paths to be biologically validated in the first instance (Fig 3, network size decrease), but also to novel alternative paths that were absent in the unweighted shortest paths analysis, and to rank more realistically the set of paths.

### Syk controls cortactin tyrosine phosphorylation via the Src tyrosine kinase

**Fig 5:**
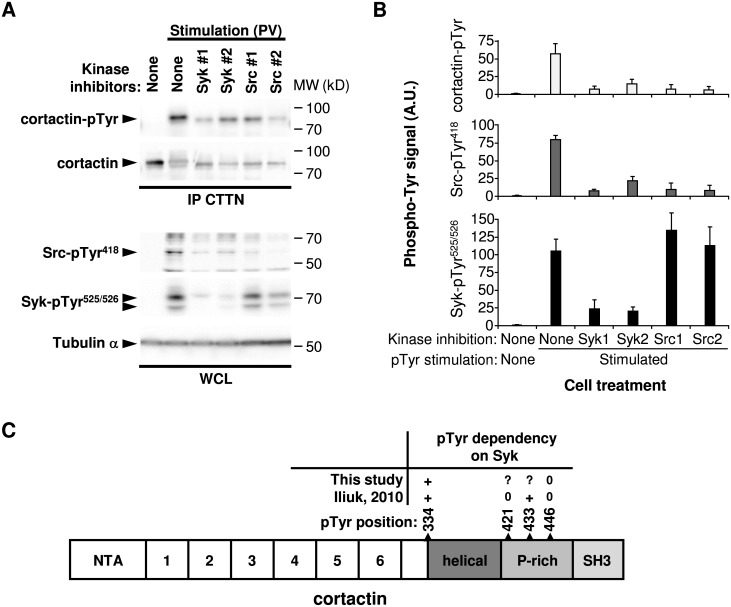
Syk controls cortactin tyrosine phosphorylation via the Src tyrosine kinase. **(A)** Phosphorylation of indicated signaling proteins (left) in MCF7 cells that were treated with kinase inhibitors (Syk1, R406; Syk2, PRT062607; Src1, PP2; Src2, AZD0530) and either left unstimulated or stimulated with pervanadate (PV). CTTN, cortactin; IP, immunoprecipitation; WCL, whole cell lysate. Molecular weight standards (MW) are indicated (right). **(B)** Protein tyrosine phosphorylation of Syk, Src and cortactin quantified after pTyr stimulation and inhibition of Syk or Src catalytic activity. In the stimulated conditions, all the values obtained with the different inhibitors are statistically significant compared to the value obtained without inhibitor (P < 0.05; n = 3), except for the Syk phosphorylation with both Src inhibitors. **(C)** Cartoon illustrating the Syk-dependent cortactin tyrosine-phosphorylation. The structure of cortactin comprises an NTA region (NH_2_-terminal acidic), 6.5 tandem repeats (37 amino acids each), a helical region (helical), a proline-rich (P-rich) region, and a C-terminal SH3 domain. The localization of the identified phosphorylated tyrosines and their dependency on Syk are indicated (+, dependent; 0, independent;?, undetermined).

Weighted shortest-path analysis from Syk to cortactin pointed to the Src tyrosine kinase as the phospho-tyrosine modifier that could account for the Syk impact on cortactin tyrosine phosphorylation (paths via white nodes in Fig 2A). This hierarchy was contradictory with previous studies describing the direct interaction of Syk and cortactin in breast cancer cells [18,42,50], and the impact of Src on Syk phosphorylation in colon cancer cells, opposite to our observations [51]. To test the ability of Src to drive the Syk signal propagation, we analyzed cortactin tyrosine phosphorylation, together with Syk and Src activity in cells treated with tyrosine kinase inhibitors. Cortactin phosphorylation and Src activity, evaluated by the phosphorylation of its tyrosine 418 residue, were decreased after cell pretreatment with Syk or Src pharmacological inhibitors (Fig 5A and 5B). Syk activity, evaluated by its auto-phosphorylation on the tyrosine 525/526 residues, was not affected by Src inhibitors, demonstrating the Syk-Src-cortactin hierarchy. In the MCF7 dataset, cortactin is poorly affected by Syk inhibition. As the quantitative measurement was obtained by calculating the median SILAC ratio of several peptides from cortactin, we decided to analyze the phosphorylation of its individual peptides. The quantity of the phosphotyrosine pTyr334 cortactin peptide was ~2 fold decreased by Syk inhibition (S8 Fig). Conversely, Tyr446 phosphorylation was not affected (S9 Fig). Those observations were consistent with the data from MDA231 dataset (Fig 5C). Taken together, our results indicate that the signal transmission from Syk to cortactin is mediated by the Src kinase.

### Syk phosphorylates ezrin on the Tyr424 residue

**Fig 6:**
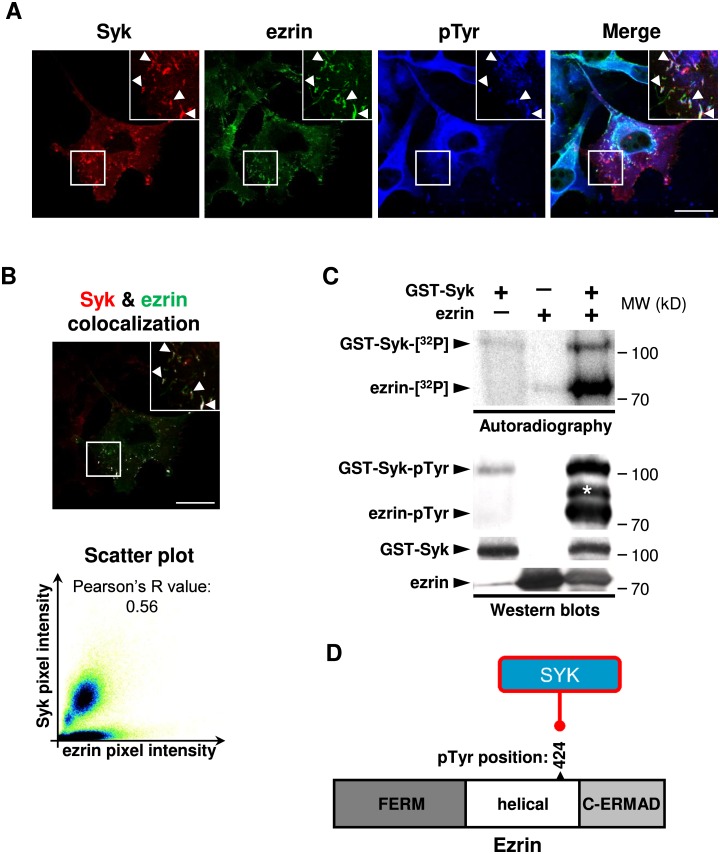
Syk phosphorylates ezrin on the Tyr424 residue. **(A)** Syk and ezrin are both localized in pTyr-enriched plasma membrane ruffles of MDA-MB-231 cells (arrowheads). Scale bar 5 μm. **(B)** Quantitative analysis of Syk and ezrin colocalization with the merged picture of Syk and ezrin channels in which colocalized pixels are displayed in white (upper panel, scale bar 5 μm) and the scatter plot of pixel intensities in Syk and ezrin channels (lower panel). **(C)** Direct *in vitro* tyrosine phosphorylation of ezrin by Syk. The phosphorylation of purified Syk and ezrin (left) are analyzed by autoradiography or Western blot (pTyr). Molecular weight standards (MW) are indicated (right). *, non-specific band. **(D)** Cartoon illustrating the ezrin phosphorylation on Tyr424 residue by Syk. The structure of ezrin comprises a NH_2_-terminal FERM domain followed by a helical domain and the C-terminal actin-binding domain (C-ERMAD).

None of the nodes directly upstream of ezrin were phospho-tyrosine modifiers that could explain the Syk impact on ezrin tyrosine phosphorylation (Fig 2B). To explore this more profoundly, we evaluated the possibility of a direct interaction between Syk and ezrin. Both proteins are localized in phospho-tyrosine enriched plasma membrane extensions (ruffles) of MDA-MB-231 breast cancer cells in which actin is dynamically reorganized (Fig 6A). Colocalization of Syk and ezrin was evaluated quantitatively (Fig 6B). Purified recombinant Syk was able to induce direct ezrin phosphorylation on tyrosine residue(s) in *in vitro* kinase assays (Fig 6C). Moreover, in an immune-complex *in vitro* kinase assay using endogenous or exogenously expressed FLAG tagged or GFP fusion-proteins, ezrin was phosphorylated dependent on the Syk catalytic activity (S10A and S10B Fig). It is worth noting that tyrosine phosphorylation of Syk is also enhanced in the presence of ezrin (compare lane 3 with lane 1) in both autoradiography and Western blot analyses which may have a biological explanation. Ezrin contains a canonical ITAM motif that has been shown to interact with Syk [52] and even play a role in Syk recruitment and activation by binding to its tandem SH2 domains [53]. A positive feedback loop may thus exist as binding of Syk is activated by its SH2 domain binding to bi-phosphorylated ITAM motifs but should be explored more deeply. Detailed analysis of the ezrin post-translational modifications by isoelectric focusing revealed that its *in vitro* phosphorylation by Syk induced a unique phosphorylation of one third of the total ezrin protein (S10C and S10D Fig). Mass spectrometric analysis demonstrated that ezrin is phosphorylated by Syk on a peptide containing the phosphorylated Tyr424 residue (S11 Fig). The same ezrin phospho-peptide was the only one identified in the Syk-positive breast cancer cells in the MDA231 dataset. Taken together, these results prompted us to propose the new protein interaction between Syk and ezrin, leading to ezrin phosphorylation on the Tyr424 residue(Fig 6D).

## Discussion

In this study, we constructed a network by which the Syk tyrosine kinase acts on its targets in breast cancer cell lines. This network is comprehensive enough to cover 350 targets, which represent 50% of the Syk targets identified in several phospho-proteomics experiments. The network was further reduced by sub-network extraction which made its analysis possible. The network enrichment and pruning steps were performed by a novel bioinformatics method that combines the shortest paths method with random walk processes. We thus propose a flexible tool, well-adapted for reconstruction and analysis of signaling networks applied on phospho-proteomic data. This method confirmed that Syk is engaged in several cancer-related pathways associated to cell growth and death, adhesion, motility, polarity and cytoskeleton regulation. In addition, it led to new biological findings concerning molecular paths in breast cancer cells linking the tyrosine kinase Syk and two targets involved in cell adhesion and motility: (i) the signaling axis Syk-Src-cortactin and (ii) the direct action of Syk on ezrin.

### Reasonability of network reconstruction and shortest path analysis

Using datasets extracted from two published complementary phospho-proteomic studies that identified Syk targets in breast cancer cells, we reconstructed a Syk-controled molecular network by integrating the components of signaling pathways enriched for Syk targets (S1 File). Two different breast cancer cell models were used: Syk-positive MCF7 cells treated or not with a pharmacological Syk inhibitor *versus* exogenous Syk expression in the Syk-negative MDA-MB-231 cells. This was expected to bring some heterogeneity in the molecular paths linking Syk to its targets. Nevertheless, lists of Syk targets emerging from the different datasets showed similarities in pathway enrichment. Moreover, in the Syk network subpart containing the reachable targets, we found that one third of the interactions involving two targets connect targets identified in different datasets, which justified combining them into a unique set despite the partial overlapping. This proportion is conserved after shortest paths analysis, suggesting that a number of mechanisms by which Syk activates its targets are common to the cell models used. Although the biochemical methods to enrich protein extracts before mass spectrometry analysis were distinct (enrichment of tyrosine phosphorylation dependent-protein complexes *versus* tyrosine-phosphorylated peptides), they produced complementary information. On the one hand, phospho-proteomic studies at the protein level identified not only proteins that are differentially phosphorylated but also their partners present in the protein complexes. Several conserved protein domains are involved in phospho-tyrosine-dependent protein-protein interaction. On the other hand, phospho-proteomic studies at the peptide level identified the differentially phosphorylated tyrosine residues, allowing to increase the precision of our experimental validations. Knowing the phosphosites provides insight in the functional consequences of the phosphorylation and the integration of this functional information allows to construct more coherent networks.

The introduction of distances was a crucial step in our approach that allowed the selection of realistic paths. The extra random walk step enabled the refinement of these distances, so the selection of too many paths having the same length was avoided. In contrast to methods proposing a unique solution (or too many alternatives), our method allows to downscale to a few biologically significant alternative paths. The simplified network obtained by sub-network extraction allowed us to generate computational hypothesis about Syk signal propagation that were subsequently validated.

### Validation of the cortactin signaling axis: Cortactin phosphorylation by Syk is dependent on Src

As a result of our biological validations, we proposed a new molecular explanation of the impact of Syk on cortactin, a protein involved in cell adhesion and motility by its ability to regulate the cortical actin cytoskeleton. Previous studies described cortactin as a direct Syk substrate [42,50]. However, in the phospho-proteomic data exploited in our study, the cortactin Tyr421 residue directly phosphorylated by Syk *in vitro* [18] does not match the Tyr residues phosphorylated in a Syk-dependent manner in cellulo [17]. In our model, we proposed a new Syk-Src-cortactin signaling axis (paths via white nodes in Fig 2A). We demonstrated that cortactin tyrosine phosphorylation induced by Syk is dependent on Src catalytic activity. Conversely, inhibition of Src did not affect the Syk catalytic activity, but both are required to induce cortactin phosphorylation in breast cancer cells (Fig 5A and 5B). This tyrosine kinase hierarchy does not match previous observations showing that Src inhibition induces a decreased tyrosine phosphorylation of Syk [51]. This discrepancy could be explained by the distinct cell models (breast cancer *versus* colorectal cancer cells) and by the fact that Leroy and colleagues [52] analyzed global Syk phosphorylation rather than specific phosphorylation on the Tyr525/526 residues, which are located in the activation loop of the Syk kinase domain and are more relevant to detect changes in Syk catalytic activity [54]. Multiple tyrosine phosphorylation sites have been described within cortactin (for review, [46]http://www.phosphosite.org/). We confirmed that the phosphorylation of cortactin Tyr446 residue is not directy affected by Syk (Fig 5C). Conversely, the phosphorylation of the Tyr334 residue is Syk-dependent, but can, according to our model, be phosphorylated also by Src [55]. There is currently no information about the functional consequences of this residue’s phosphorylation. Finally, the Syk-Src-cortactin signaling axis we proposed is consistent with the positive impact of Syk on E-cadherin dependent cell-cell adhesion [19,42]. Src-dependent phosphorylation of cortactin is necessary to link the E-cadherin adherens junction complex to the actin cytoskeleton, that subsequently supports cell-cell contact formation [56,57].

### Validation of the ezrin signaling axis: Syk directly phosphorylates ezrin

Our initial computational hypotheses of molecular circuits linking Syk to ezrin did not explain its tyrosine phosphorylation, by the lack of a phospho-tyrosine modifier directly upstream of ezrin (Fig 2B). We demonstrated that Syk phosphorylates ezrin in a direct manner on its Tyr424 residue and that both proteins colocalize in the plasma membrane ruffles in breast cancer cells (Fig 6A and 6B). Previous studies described a Syk-dependent tyrosine phosphorylation of ezrin in B lymphocytes on its Tyr353 residue [58,59], a site that can be also phosphorylated by the epidermal growth factor receptor (EGFR) [60]. Phosphorylation of this residue leads to activation of the ezrin downstream signaling pathways as JNK or PI3K/Akt [59,61]. Two other ezrin tyrosine residues, Tyr146 and Tyr478, can be phosphorylated by Src (Tyr146, also being phosphorylated by EGFR), mediating cell scattering and stimulating motility [62,63]. We showed here that in breast cancer cells, Syk phosphorylates ezrin on the Tyr424 residue, rather than Tyr353 as it occurs in B lymphocytes, and could induce a signaling cascade different than the ones previously described for tyrosine phosphorylated ezrin. This novel molecular regulation of ezrin could help to explain the negative impact of Syk on epithelial cell motility.

### Interest for cancer research and extent of application of the methods

By this study, we do provide to the cancer cell signaling community access to the Syk network and sub-networks of the paths from Syk to its targets involved in (**i**) cell adhesion and motility, (**ii**) cell growth and death, (**iii**) cell differentiation and (**iv**) immunity and inflammation (see Supporting Information). As Syk is involved in breast cancer suppression, it is not surprising that these cellular processes involved in cancer progression can be affected by Syk in breast cancer cells. Nonetheless, many of the Syk interactions described in pathway databases are extracted from molecular studies in cells of hematopoietic origin. More precisely, Syk signaling has been extensively studied in B lymphocytes where it is indispensable for immune cell differentiation. Which part of Syk signaling is shared between epithelial and hematopoietic cell types could be determined by Syk related phospho-proteomic experiments with a more comprehensive collection of cell models.

At this stage, our model suggests a number of plausible mechanisms linking Syk with cancer-related cellular processes. These can be used to generate more hypotheses, validation and provide valuable inputs for further developments. A crucial issue in Syk-related research is how to activate compensatory mechanisms maintaining tumor suppression signaling even when Syk is downregulated. The network we propose here is the first step towards addressing this question. To advance towards more refined mechanistic models the annotation of the interactions, for instance the sign, should be completed by integration of more data and by formal inference methods [64,65]. The careful consideration of possible feed-backs should also be considered in these developments. For instance, in tyrosine kinase signaling, many feed-back interactions involve phosphatase players whose role and significance are largely ignored even for very well studied pathways such as MAPK. New biological experiments are needed to unravel new players and interactions. Basic networks like the one resulting from our approach can be used for planning such experiments. As a simple example, if phosphatases are present upstream in the network one would want to test the effect of their inhibition on downstream proteins.

The bioinformatics method we used to reconstruct and prune the Syk signaling network can be used in other studies, whenever the focus is on finding candidate mechanisms explaining how signals propagate in large networks and how the network state-changes under perturbations. We consider that the combination of *ad-hoc* distances, random walk, and near-shortest paths provides good candidate mechanisms, by implementing the following requirements: (i) minimize the number of steps (shortest paths); (ii) fit with the data (*ad-hoc* distances); (iii) further favor intermediates which belong to multiple appropriate candidate paths (random walk); (iv) propose and rank multiple alternatives (near-shortest paths). This strategy can be generally applied to other studies of signaling networks using datasets based on distinct post-translational modifications, separately or combined. The open source code for the network reconstruction and extraction of relevant sub-networks has been made available for the computational biology community (S1 File and https://github.com/aurelien-naldi/NetworkReconstruct).

## Materials and methods

### Online databases

Uniprot ID mapping from uniprot.org/downloads (2015/07)

HGNC dataset from genenames.org/cgi-bin/statistics (2015/07)

GO ontology from geneontology.org/page/download-ontology (go-basic.obo, 2015/10)

GO annotation from geneontology.org/page/download-annotations (goa_human.gaf, 2015/10)

KEGG: www.kegg.jp, release 75 (2015/07)

Pathway commons:pathwaycommons.org release 7 (2015/03)

### Mapping protein identifiers

Proteins are identified by their Uniprot IDs, without the isoform postfix. We used Uniprot ID mapping files to associate KEGG and HGNC identifiers (transferred to the corresponding gene symbols) with these Uniprot IDs. When multiple Uniprot IDs are associated to the same KEGG or HGNC ID, they were grouped and a single ID is selected for the group, preferably a reviewed entry (allowing to map unreviewed Uniprot entries to the associated reviewed entry when possible).

### Annotation of Syk network

The network is annotated based on the dataset, the pathways, and the GO annotation. Nodes and interactions keep track of the list of pathways in which they appear. Interactions types (phosphorylation, modification, regulation) and signs (+ or -) are transferred from the pathways when available. When the same interaction is described in several pathways, all types and a combination of the signs are conserved (an interaction described as positive in a pathway and negative in another is considered as unclear). We selected some groups of GO terms representing relevant processes and functions in this dataset, in particular cell adhesion and motility (GO:0048870, GO:0007155, GO:0034330, GO:0022610, GO:0060352, GO:0030030), cell growth and death (GO:0008283, GO:0007049, GO:0008219, GO:0019835, GO:0000920, GO:0007569, GO:0051301, GO:0060242), immunity and inflammation (GO:0002376, GO:0001906), and cell differentiation (GO:0030154, GO:0036166). We also annotated as phospho-tyrosine modifiers the components of the network with tyrosine kinases (GO:0004713) and tyrosine phosphatases (GO:0004725) GO terms and manually verified this list (S7 Table).

Many nodes in KEGG pathways represent groups of proteins, where the same protein can be part of several groups (with variable overlap). These groups are conserved in the merged network by introducing “group nodes” with bidirectional links to their members. Proteins can thus have interactions associated directly to them or through one or several groups. Such groups are “exploded” before the path search step described below.

### Network visualization

Cytoscape 3.4 (http://www.cytoscape.org/) was used to generate figures and Cytoscape web was used to provide interactive access to the Syk network.

### Near-shortest paths

We searched for “near-shortest paths” between Syk and a list of targets, using an approach similar to the classical Dijkstra algorithm for shortest paths. We started by identifying the length of the shortest path for every node as in Dijkstra’s method (starting from the source node, we iteratively picked the closest new neighbor of all reachable nodes: at each step we obtained the best result for a new node, ending with the node with the longest of the shortest paths). In the Dijkstra algorithm, the shortest paths were then obtained by starting from the target nodes and going backward to the source by selecting the incoming edge(s) which can satisfy this best distance: i.e. the best distance of the current node is equal to the sum of that of the source and the distance of the edge. Here we define an acceptable extra distance to include additional nodes and edges during this backtracking step. Note that the resulting sub-network can contain paths that are longer than acceptable, but all selected nodes and edges are involved in at least one acceptable path. For example if (A,B,C) is the shortest path from A to C, and (A,I,B,C) and (A,B,J,C) are also acceptable, then the path (A,I,B,J,C) exists in the resulting sub-network despite being too long. In the resulting sub-network, the selected nodes and edges are annotated with the “overflow” needed to include them: i.e. the extra distance of the best path using them compared to the actual shortest path. Members of the shortest paths have no overflow.

### Edge distances for the shortest paths

To improve the identified paths, we selected edge weights based on the available annotations: “normal” edges have a distance of 5 (d = 5), we promoted edges coming out of identified proteins (d = 3), edges reaching an identified protein while coming out of a tyrosine kinase or phosphatase (d = 2) or combining these two conditions (d = 1). On the other end, we demoted edges reaching a target identified as differentially phosphorylated, but which did not come from a tyrosine kinase or phosphatase (d = 8), even if they come out of another identified protein (d = 6). Finally, edge distances are refined to integrate the results of the random walk estimation: they are multiplied by the inverse of the normalized score of their source node.

### Random walk

We adapted the netwalk implementation in R from the GUILD software [66]: http://sbi.imim.es/web/index.php/research/software/guildsoftware

Edge weights are based on the distances originally defined for the shortest paths search (reversed as a higher distance corresponds to a lower weight).

More precisely, let *d*_*ij*_ be the distance from a node *i* to its direct target *j*, previously defined for the shortest path search. Given a node *i* the set of all its direct targets (successors in the directed network) is denoted *Succ(i)*. The random walk is defined by transition probabilities *p*_*ij*_ defined as:

$$p_{ij}=\frac{(1-p_{0})d_{ij}^{-1}}{\sum {}_{j\in Succ(i)}d_{ij}^{-1}},$$

where *p*_*0*_ is the return probability to the origin node Syk (chosen the same for all nodes).

The probabilities *p*_*ij*_ together with *p*_*0*_ added as last entry of each row are the entries of the stochastic matrix ***P*** (each row of this matrix sums to one). The equilibrium or limiting distribution of the random walk is a normalized row vector ***π*** satisfying the equation:

πP=π.

This distribution can be estimated by starting the random walk from any node and running it a sufficiently long time for equilibration. A finite, connected network with possibility of return to the Syk node from terminal nodes is ergodic guaranteeing the existence and uniqueness of the equilibrium distribution.

Nodes are scored by the values of the equilibrium probabilities *π*_*i*_. In order to eliminate biases created by topology a second simulation is performed where all edges have the same weight. The resulting scores in this second simulation are the equilibrium probabilities $\pi_{i}^{0}$. The two scores are then used to refine the distances as follows

$${\tilde{d}}_{ij}=d_{ij}\frac{\pi_{i}^{0}}{\pi_{i}}.$$

### Cell culture

MCF7, MDA-MB-231 and COS7 cell lines were obtained from the ATCC and maintained in Dulbecco’s modified Eagle’s medium (DMEM, Gibco) supplemented with 10% fetal calf serum (FCS, Eurobio). All cell cultures were carried out at 37°C using a 5% CO2 atmosphere. For cell stimulation studies, cell lines were stimulated with Sodium pervanadate (PV, premix of 1 mM H_2_O_2_ and 1 mM Na_3_VO_4_) and incubated for 15 min at 37°C. For the evaluation of the effect of the kinase inhibitors (all from Selleckchem), cells were incubated in medium for 2 hours with 2.5 mM R406, 5 mM PRT062607, 1 mM PP2, 0.5 mM AZD0530. Stock solutions for all those kinase inhibitors were prepared in dimethyl sulfoxide (Sigma, Hybri-Max grade), which is used as vehicle negative control

### Western blot analyses

Cells were washed with ice-cold phosphate buffered saline solution and scrapped in 10 mM Tris-HCl (pH 7.4), 150 mM NaCl, 0.5 mM EDTA (Sigma), 1% Nonidet-P40 (Sigma), 0.5% sodium deoxycholate (Sigma), 1 mM Na_3_VO_4_ (Sigma), 50 mM NaF (Sigma) and a protease inhibitor cocktail (Sigma) at 4°C. After transfer to an Eppendorf tube and extensive vortexing, lysates were cleared by centrifugation at 10,000 rpm for 10 min at 4°C and supernatants diluted in 4x SDS-PAGE sample buffer. Immunoprecipitations were performed as described previously [44]. Protein samples were diluted in 4x SDS-PAGE Laemmli sample buffer, denatured by boiling for 5 min at 95°C in SDS-PAGE sample buffer, separated electrophoretically and transferred to polyvinylidene difluoride membranes. Membranes were blocked using 5% BSA in tris-buffered saline solution with Tween-20 detergent (TBS-T; 25 mM Tris-HCl pH 8.0, 150 mM NaCl, 0.1% Tween-20) for 1 h and then incubated at 4°C with the appropriate primary antibodies diluted in blocking buffer. Those included a mix of two monoclonal antibodies to phospho-tyrosine (1:1 mix vol/vol mix of the 4G10 and PY20 hybridoma supernatants), monoclonal antibodies to the FLAG epitope (clone M2, Sigma), cortactin (clone 4F11; Millipore), Syk (clone 4D10, Santa Cruz) and alpha-tubulin (clone DM1A, Sigma), rabbit polyclonal antibodies to pTyr^418^ Src (Invitrogen), GFP (Chemokine), a home-made rabbit polyclonal antibody to the COOH-terminal domain of human ezrin [67], and a rabbit monoclonal antibody to pTyr525/526 Syk (Cell Signaling Technology). After three washes with TBS-T, the membrane was incubated with horseradish peroxidase-conjugated appropriate secondary antibody (1:5000, Jackson ImmunoResearch) for 1 h at room temperature. Immunoblots were revealed using a standard chemoluminescent method (ECL, Ozyme) and a Multi-application gel imaging system (PXi, Syngene). Membranes were optionally stripped with the Restore PLUS Western blot stripping buffer (Thermo Scientific) before a second immunoblotting. Immunoblot-derived signals were quantified using the ImageJ software (NIH) with three independent biological and technical replicates for each quantification. The signals were normalized on the lane corresponding to the total protein quantity loaded (immunoglobulin heavy chain in case of immunoprecipitation; Tubulin-α in case of whole cell lysate) and the unstimulated condition was arbitrarily set at 1.

### In vitro phosphorylation assay

Assays with proteins extracted from cell lysates, immunoprecipitations and Western blot analyses were performed as described previously [43]. Otherwise, recombinant GST-Syk (BPS Bioscience, San Diego, CA) and GST-ezrin (previously described in [68]) were used. *In vitro* kinase assays were performed as described [43]. For the two-dimensional electrophoresis analysis, proteins were precipitated for 2 h in two volumes of acetone at −20°C, and resuspended in 8 M urea, CHAPS 4%, and thiourea 2 M. We used 18 cm IPG-strips (Amersham Biosciences) with linear pH range of 3–10 for the first dimension. Proteins were loaded on the IPG-strips and run in a Multiphor II apparatus (Amersham Biosciences). After focusing, a second migration was performed in 10% SDS-PAGE gel and proteins were stained with silver nitrate (Amersham Biosciences).

### Microscopy

MDA-MB-231 cells were transiently transfected with pDsRed-Syk [43] using Fugene 6 (Roche Applied Bioscience). Immunostaining procedures have been described before [43]. The following primary antibodies were used: monoclonal antibody 4G10 hybridoma supernatant (diluted 1:50 in TBS) and a home-made rabbit polyclonal antibody to the C-terminal domain of human ezrin [67]. The secondary antibodies used were goat-anti-mouse-Cy5 and donkey-anti-rabbit-FITC (Jackson ImmunoResearch Laboratories). Confocal images of immunostained cells were obtained as described [69]. For quantitative analysis of colocalization, we used the ImageJ software plug-ins (https://imagej.nih.gov/ij/) with the “Colocalization Finder” module (https://imagej.nih.gov/ij/plugins/colocalization-finder.html) to generate the merged picture of Syk and ezrin channels in which colocalized pixels are displayed in white, and with the “Coloc 2” module (http://imagej.net/Coloc_2) to generate the scatter plot of pixel intensities in Syk and ezrin channels and to compute the Pearson correlation of pixel intensities over space.

### Proteomic analyses

#### Sample preparation

Proteins were separated by SDS-PAGE and the gel was stained with the Colloidal Brillant Blue G (Sigma). Protein bands were in-gel digested using trypsin (sequencing grade; Promega, Charbonnières, France) [70].

#### Tandem mass spectrometry

Analysis of the samples was performed on a QSTAR pulsar-i quadripole-time-of-flight mass spectrometer (Applied Biosystems, Foster City, USA) coupled to an Ultimate 3000 (Dionex, Amsterdam, Netherland) nanoflow system driven by Chromeleon software. A gradient consisting of 0–40% B in A for 60 min, 80% B in A for 15 min (A = 0.1% formic acid, 2% acetonitrile in water; B = 0.1% formic acid in acetonitrile) flowing at 300 nl/min was used to elute peptides from the capillary (75 μm x 150 mm) reverse-phase column (Pepmap, Dionex). Desalting and pre-concentration of samples were achieved on-line on a Pepmap precolumn (300 μm x 10 mm). All MS spectra were acquired in data-dependent mode using Analyst QS 1.1 software. Briefly, the mass spectrometer was operated in the information-dependent acquisition mode to automatically switch between MS and MS/MS acquisition. Survey full-scan TOF-MS spectra (from m/z 350–1600) were acquired during 1 s. The two most intense ions were sequentially isolated and fragmented using collisional-induced dissociation. Each MS/MS spectrum was acquired during 3 s and the precursor was excluded during 45 s. Parameters were adjusted as follows: ion spray voltage (IS), 1800 V; curtain gas (CUR), 25; declustering potential (DP), 60 V; focusing potential (FP), 265 V; declustering potential 2 (DP2), 15 V. Peptide fragmentation was performed in the collision cell using nitrogen gas on the doubly, triply, or quadruply charged ions detected, with a collision energy set “on-the-fly” using the rolling collision energy feature based on their charge and mass.

#### Database analysis

MS/MS spectra were searched against the human entries of UniProt Knowledgebase Release 10.2 database (http://www.expasy.ch) by using the Mascot v2.1 algorithm (http://www.matrixscience.com). Search parameters were mass accuracy 0.1 Da for MS and MS/MS data; 1 miscleavage; variable modifications: oxidized methionine, Phospho-Ser/Thr, Phospho-Tyr, SILAC-labels: Lys-8 and Arg-10. All significant hits (p<0.05) were manually inspected.

#### Quantification

Quantification was done on at least two MS spectra per protein by using MSQuant v1.4.1 software developed by Mann and colleagues [71] (http://msquant.sourceforge.net). Data were manually inspected and corrected when necessary.

#### Post-treatment of phospho-proteomic data

The first dataset was obtained after inhibition of Syk catalytic activity in Syk-positive MCF7 cells [19]. Proteins were purified on an anti-phospho-tyrosine affinity column, and enriched phospho-tyrosine-dependent complexes were identified by mass spectrometry. The SILAC (Stable Isotope Labeling with Amino acids in Cell culture) strategy allows relative quantification of protein ratio between control and Syk-inhibited cells. As the Syk inhibitor concentration only partly inhibits the Syk catalytic activity, we selected proteins with a SILAC ratio reflecting a variation of 10% as Syk targets. On 479 proteins identified, 240 proteins exhibited an increased phosphorylation or association with phosphoproteins in presence of full Syk activity, 25 proteins in presence of inhibited Syk activity (S1 Table). The second dataset was obtained after Syk expression in Syk-negative MDA-MB-231 cells [17]. In this study, proteins were trypsin-digested prior to two steps purification on an anti-phospho-tyrosine affinity column and on polymer-based metal ion affinity column. We compared the tyrosine-phosphorylated peptides identified in Syk-positive and Syk-negative cells by Iliuk and colleagues (2010). Phospho-peptides identified in only one experimental condition were considered as differentially phosphorylated (461 in Syk-positive cells and 125 in Syk-negative cells). Phospho-peptides identified in Syk-positive cells match to 385 proteins, those identified in the Syk-negative cells to 117 proteins. Amongst them, 15 proteins displayed distinct phospho-peptides identified in both experimental conditions. Due to amino acid sequence redundancy, 25 of the differentially phosphorylated peptides matched to several proteins that we all included in the differentially phosphorylated proteins. We considered all these proteins as Syk targets. 131 phospho-peptides were found in both experimental conditions, including 9 with the same phospho-tyrosine residue but with a differential methionine oxidation, and were excluded (S2 Table). The third dataset identified the peptides phosphorylated on tyrosine by Syk after *in vitro* kinase reaction [18]. Amongst them, we selected as Syk direct substrates those that were also identified in the MDA-MB-231 cells after Syk expression by Iliuk and colleagues (2010). Contrary to the list published by Xue and colleagues (2012), we excluded the following proteins because their phospho-peptides identified *in vitro* were not retrieved exclusively in Syk expressing MDA-MB-231 cells: Four and a half LIM domains protein 2 (Uniprot #Q14192), Tyrosine-protein phosphatase non-receptor type 11 (Uniprot #Q06124), Tyrosine-protein phosphatase non-receptor type 1 (Uniprot #P18031), cortactin (Uniprot #Q14247), Vimentin (Uniprot #P08670) and Tyrosine-protein phosphatase non-receptor type 12 (Uniprot #Q05209). We also excluded the ITSFPESEGYSyETSTK phospho-peptide of the MAP1B protein (Uniprot #P46821) for the same reasons (but the MAP1B protein remains as a Syk direct substrate because another MAP1B phospho-peptide was retrieved exclusively in Syk-expressing MDA-MB-231 cells). Finally, we included the Cofilin-1 and Cofilin-2 proteins (Uniprot #P23528 and #Q9Y281, respectively) because the YALYDATyETK phospho-peptide was retrieved exclusively in Syk-expressing MDA-MB-231 cells (S2 Table).

### Statistical analysis

Statistical analyses were performed using the two-tailed Student’s t test for paired and unpaired data *versus* control values. Experimental values in this work are all given as mean and standard error of the mean (SEM). Results with a P value ≤ 0.05 were considered as statistically significant.

## Supporting information

S1 Table MCF7 dataset. Dataset of Syk targets in breast cancer cells from Larive et al. (2009). “+”, proteins with an increased phosphorylation or association with phospho-proteins in presence of active Syk; “-”, proteins with a decreased phosphorylation or association with phospho-proteins in presence of active Syk. Proteins from both situations are considered as Syk targets.
(ODS)

S2 Table MDA231 dataset. Datasets of Syk targets in breast cancer cells from Iliuk et al. and Xue et al. “+”, phospho-peptides identified in Syk-positive cells; “-”, phospho-peptides identified in Syk-negative cells. Proteins identified with peptides from both situations are considered as Syk targets.
(ODS)

S3 Table Network components selected as phospho-tyrosine modifiers with tyrosine kinases (GO:0004713) and tyrosine phosphatases (GO:0004725) GO terms and manually verified. (ODS)

S4 Table Enriched pathways in the lists of Syk targets from MCF7 dataset, using pathways from the KEGG database. (ODS)

S5 Table Enriched pathways in the lists of Syk targets from MDA231 dataset, using pathways from the KEGG database. (ODS)

S6 Table Enriched pathways in the lists of Syk targets from MCF7 and MDA231 datasets, using pathways from the KEGG database. (ODS)

S7 Table Enriched pathways in the lists of Syk targets from MCF7 and MDA231 datasets, using pathways from the Pathway Commons database. (ODS)

S1 Fig Network showing all selected paths from Syk to identified targets. The color of nodes represents associated GO annotations: red for cell adhesion and motility, green for cell growth and death, blue for immunity and inflammation. Proteins associated to several groups have composed colors. Black nodes are associated with all groups, grey ones with none. The larger squares highlight proteins found in the original datasets. Syk is the largest node.
(PDF)

S2 Fig Evolution of the sub-network for the effect of Syk on proteins associated to cell growth and death using unweighted shortest paths, after the integration of weights (A), and after refinement using random walks (B). Network elements are annotated as Fig 3.
(PDF)

S3 Fig Evolution of the sub-network for the effect of Syk on proteins associated to cell differentiation using unweighted shortest paths, after the integration of weights (A), and after refinement using random walks (B). Network elements are annotated as Fig 3.
(PDF)

S4 Fig Evolution of the sub-network for the effect of Syk on proteins associated to immunity and inflammation using unweighted shortest paths, after the integration of weights (A), and after refinement using random walks (B). Network elements are annotated as Fig 3.
(PDF)

S5 Fig Evolution of the sub-network for the effect of Syk on proteins associated to cell growth and death using weighted shortest paths, and after refinement with random walks, both allowing a 20% overflow. Network elements are annotated as Fig 4.
(PDF)

S6 Fig Evolution of the sub-network for the effect of Syk on proteins associated to cell differentiation using weighted shortest paths, and after refinement with random walks, both allowing a 20% overflow. Network elements are annotated as Fig 4.
(PDF)

S7 Fig Evolution of the sub-network for the effect of Syk on proteins associated to immunity and inflammation using weighted shortest paths, and after refinement with random walks, both allowing a 20% overflow. Network elements are annotated as Fig 4.
(PDF)

S8 Fig Effect of Syk on the phosphorylation of cortactin pTyr334 residue. **(A)** MS spectrum of the cortactin heavy and light peptides containing the phosphorylated Tyr 334 residue and showing their relative abundance in pervanadate-activated MCF7 cells pretreated or not with Syk inhibitor (Pic, piceatannol). **(B)** MS/MS identification of the cortactin heavy peptide containing the phosphorylated Tyr334 residue.
(PDF)

S9 Fig Effect of Syk on the phosphorylation of cortactin pTyr446 residue. **(A)** MS spectrum of the cortactin heavy and light peptides containing the phosphorylated Tyr 446 residue and showing their relative abundance in pervanadate-activated MCF7 cells pretreated or not with Syk inhibitor piceatannol (Pic). **(B)** MS/MS identification of the cortactin heavy peptide containing the phosphorylated Tyr 446 residue.
(PDF)

S10 Fig Syk controls ezrin tyrosine phosphorylation. **(A)** After protein extraction from MCF7 cells and Syk and ezrin protein immunoprecipitation (IP), the *in vitro* kinase reaction is performed with [^32^P]-ATP either in the presence or absence of Syk inhibitor piceatannol (PIC). **(B)** COS7 cells are expressing FLAG-Syk (1), ezrin-GFP (2), both (4) or ezrin-GFP and FLAG-Syk kinase dead (KD) mutant (lane 3). After cell lysis and immunoprecipitation (IP) with the indicated antibodies (bottom), the *in vitro* kinase reaction is performed with [^32^P]-ATP. **(C-D)** COS7 cells expressing FLAG-Syk and ezrin-GFP are lysed, proteins are immunoprecipitated and the *in vitro* kinase reaction is performed in presence or absence of ATP. Proteins are then incubated either with alkaline phosphatase or not. Part of the reaction product is analyzed for tyrosine phosphorylation of ezrin after SDS-PAGE **(C)**. Part of the reaction is analyzed by two-dimensional gel electrophoresis **(D)**. Arrow designs the phosphorylated ezrin. IEF, isoelectro focusing.
(PDF)

S11 Fig MS/MS identification of the ezrin peptide containing the phosphorylated Tyr 424 residue. (PDF)

S1 File Python code tools for all the steps described in the paper. Data representing the full Syk network and the subnetworks analyzed in the paper. An example of web visualization of the Syk networks based on a customized Cytoscape Web.
(ZIP)
