# Supplementary figures and images for: Reconstruction and signal propagation analysis of the Syk signaling network in breast cancer cells
Source: PLoS Comput Biol. 2017 Mar 17;13(3):e1005432. doi: 10.1371/journal.pcbi.1005432 (PMC5376343; doi:10.1371/journal.pcbi.1005432)

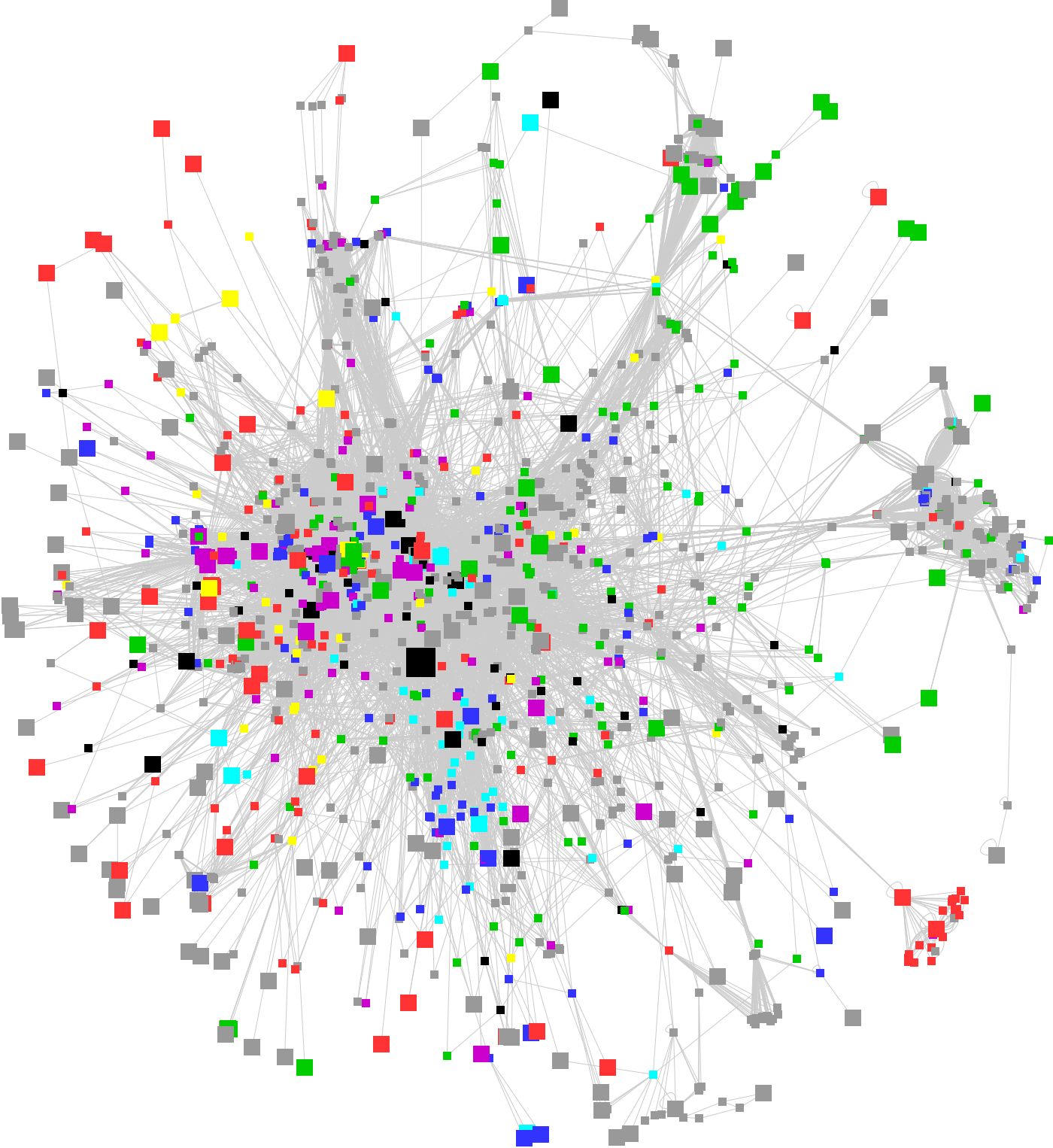

Supplement: S1 Fig — The color of nodes represents associated GO annotations: red for cell adhesion and motility, green for cell growth and death, blue for immunity and inflammation. Proteins associated to several groups have composed colors. Black nodes are associated with all groups, grey ones with none. The larger squares highlight proteins found in the original datasets. Syk is the largest node. (PDF) [file pcbi.1005432.s008.pdf]

**A****Shortests** → **Weighted**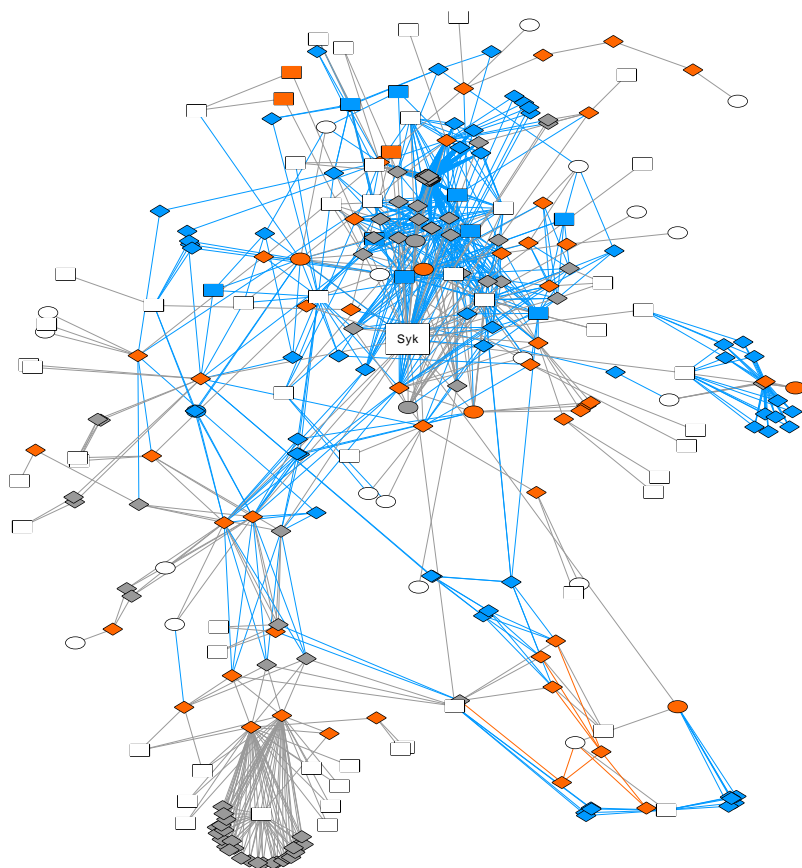**B****Weighted** → **Refined**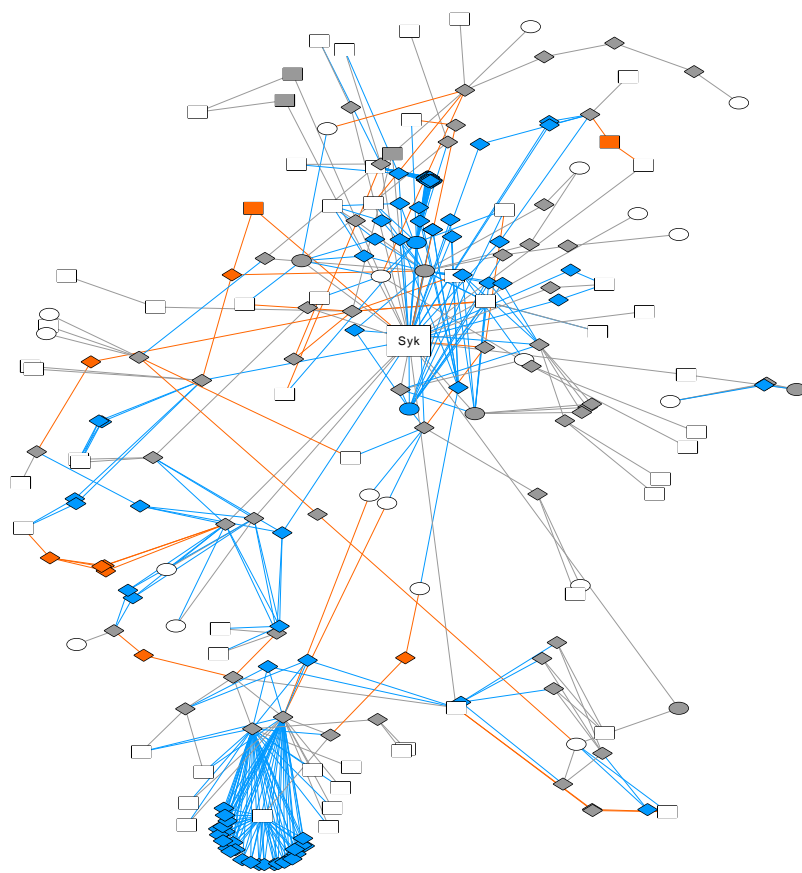

Supplement: S2 Fig — Network elements are annotated as Fig 3. (PDF) [file pcbi.1005432.s009.pdf]

**A****Shortests** → **Weighted**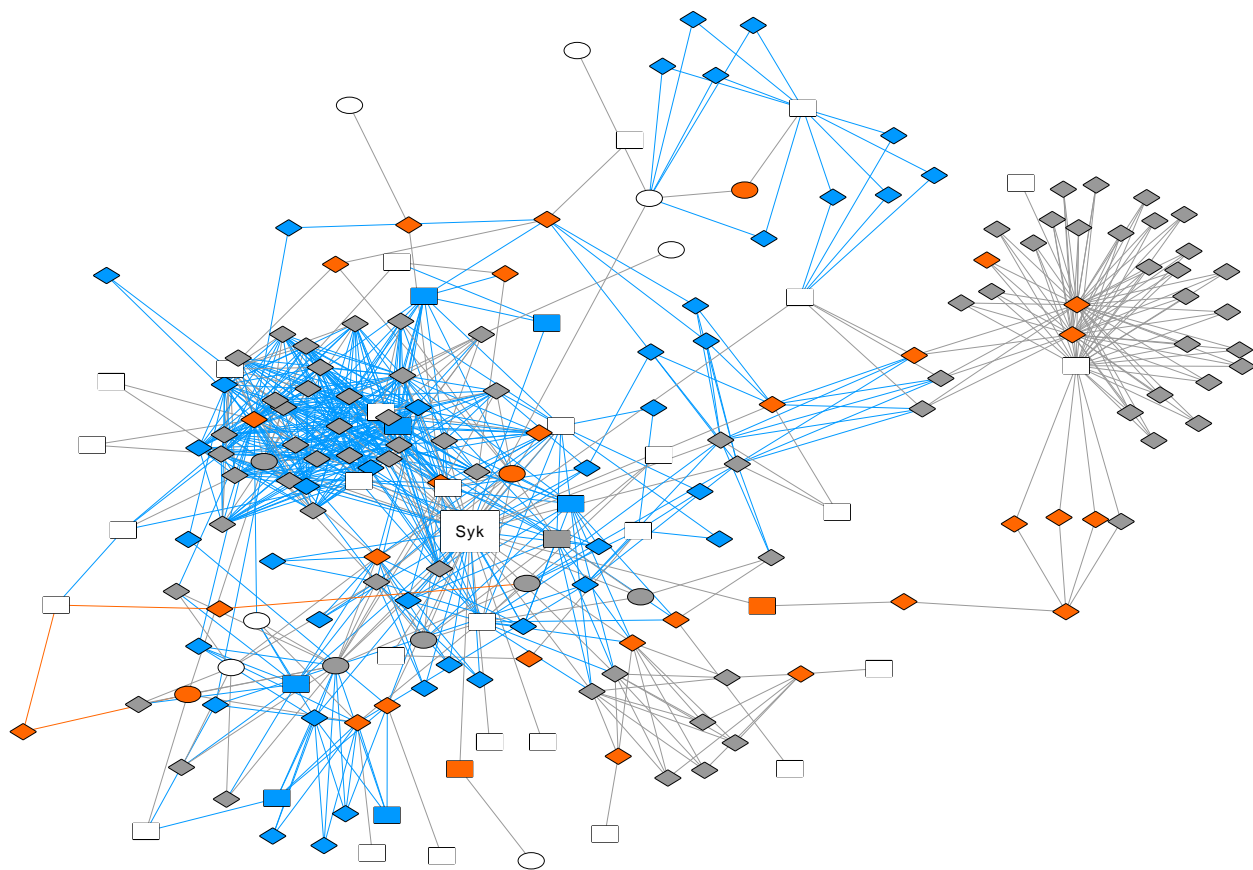**B****Weighted** → **Refined**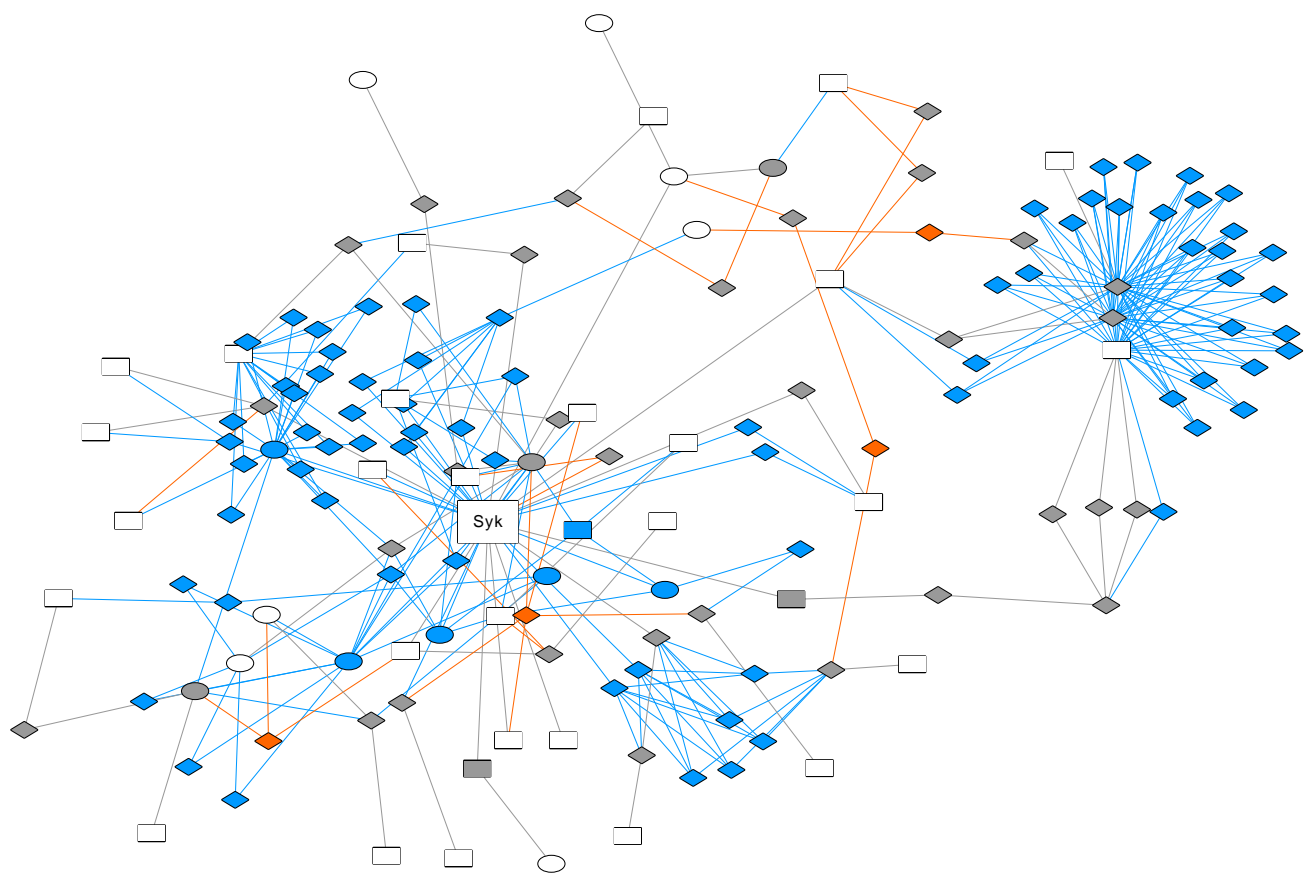

Supplement: S3 Fig — Network elements are annotated as Fig 3. (PDF) [file pcbi.1005432.s010.pdf]

**A****Shortests** → **Weighted**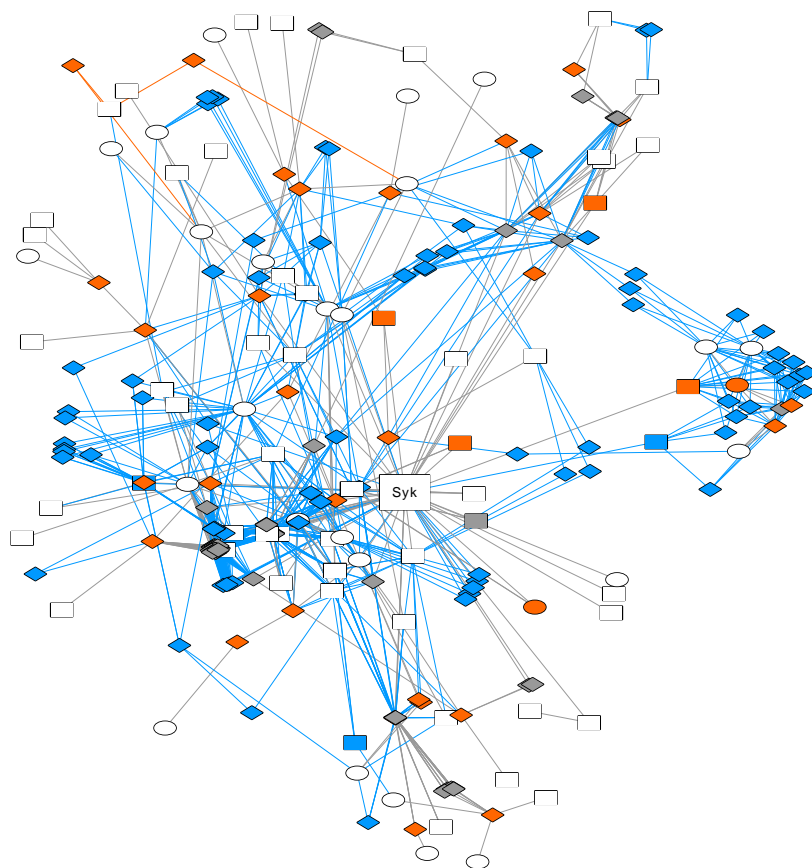**B****Weighted** → **Refined**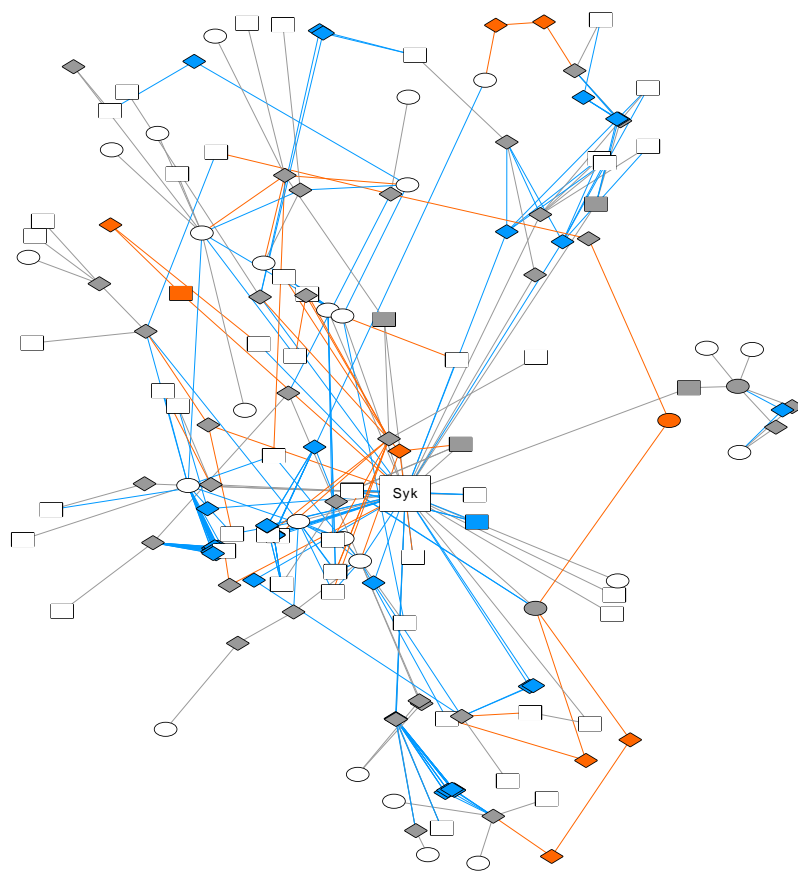

Supplement: S4 Fig — Network elements are annotated as Fig 3. (PDF) [file pcbi.1005432.s011.pdf]

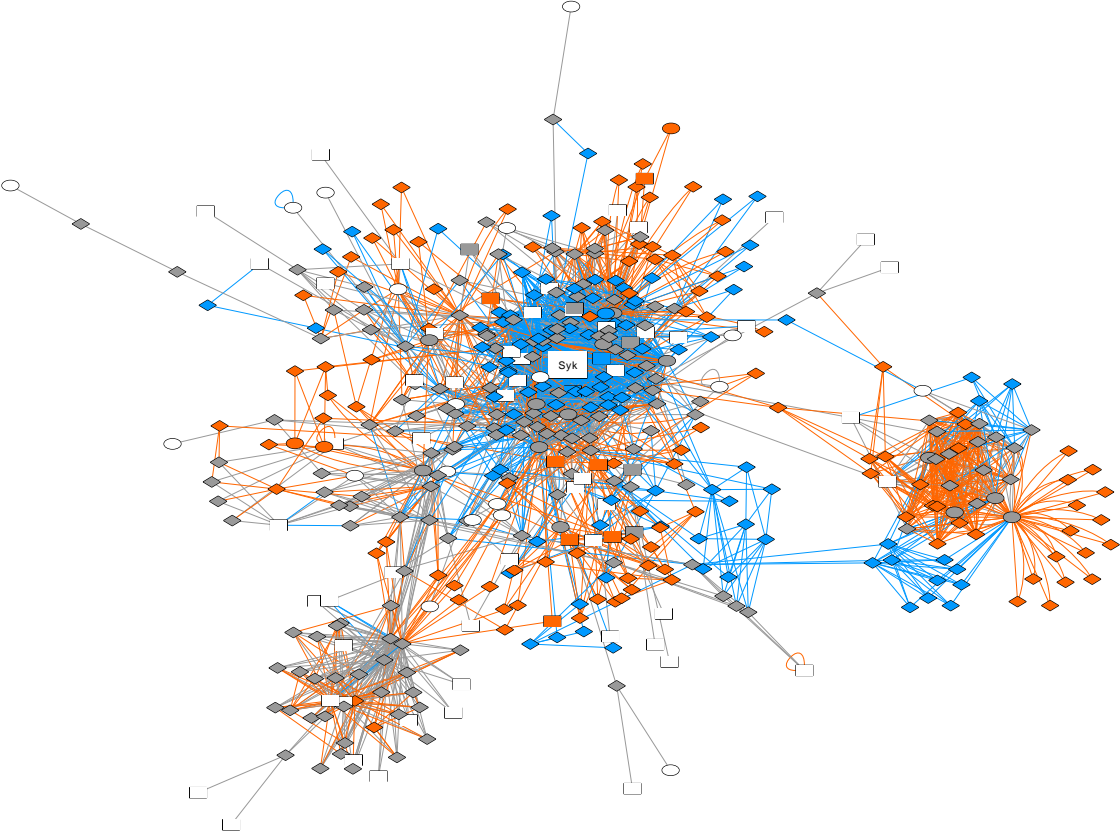

Supplement: S5 Fig — Network elements are annotated as Fig 4. (PDF) [file pcbi.1005432.s012.pdf]

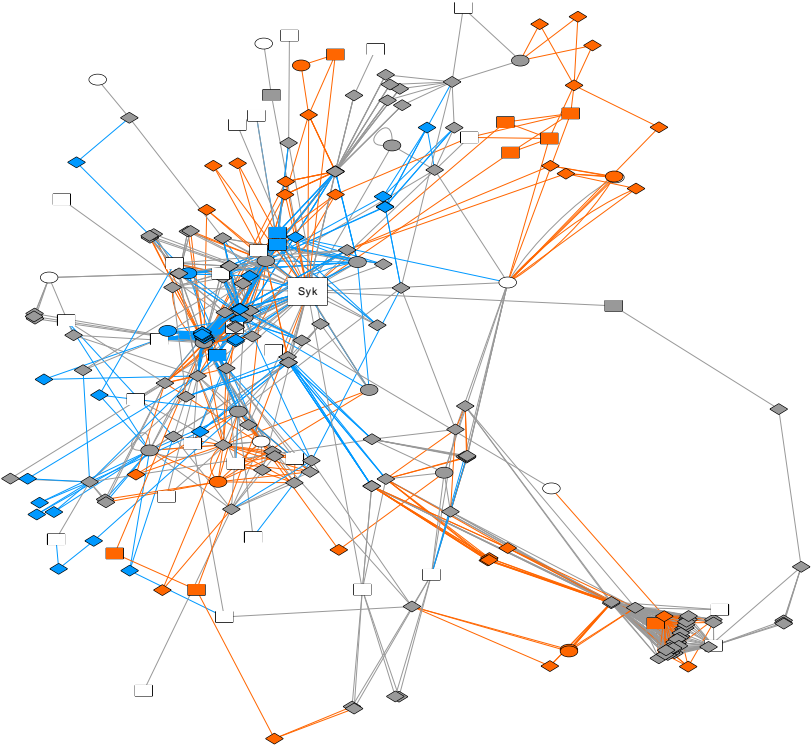

Supplement: S6 Fig — Network elements are annotated as Fig 4. (PDF) [file pcbi.1005432.s013.pdf]

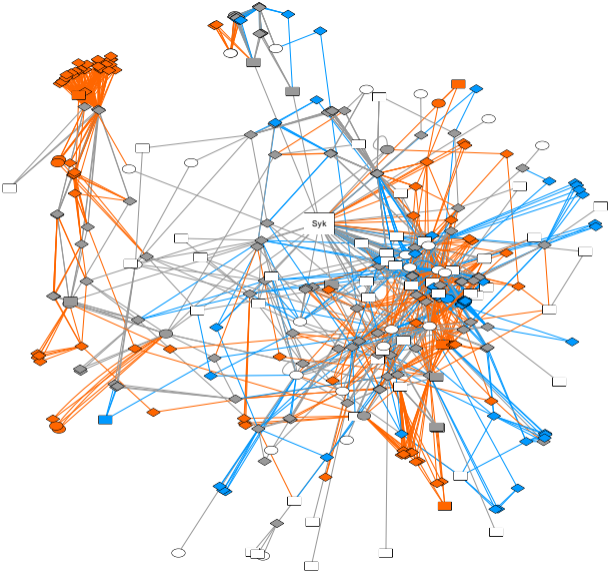

Supplement: S7 Fig — Network elements are annotated as Fig 4. (PDF) [file pcbi.1005432.s014.pdf]

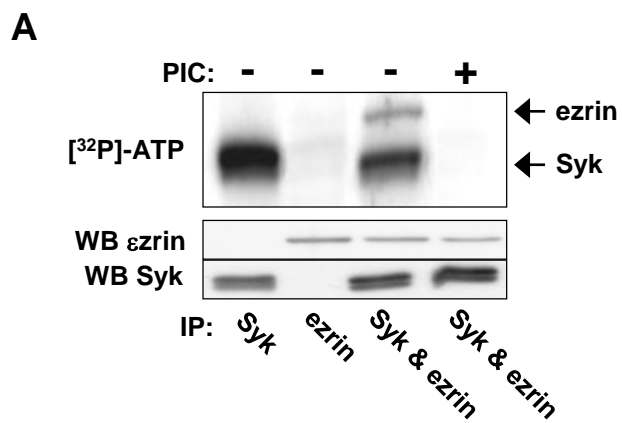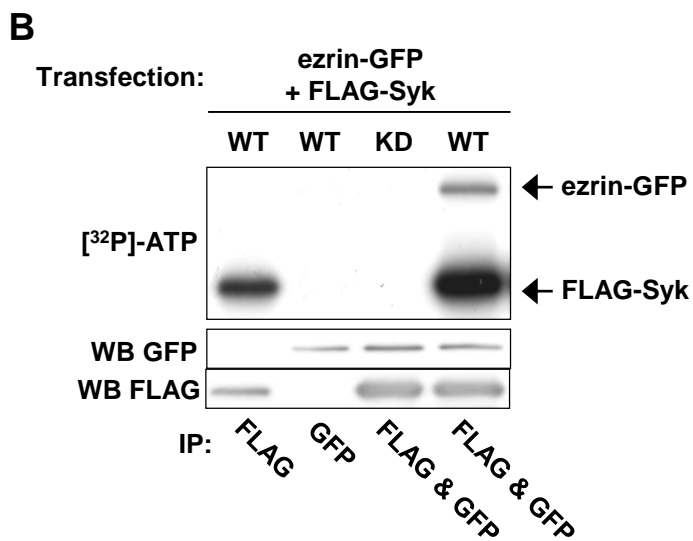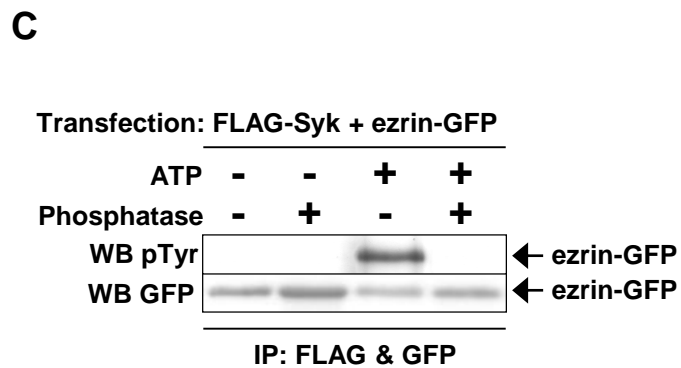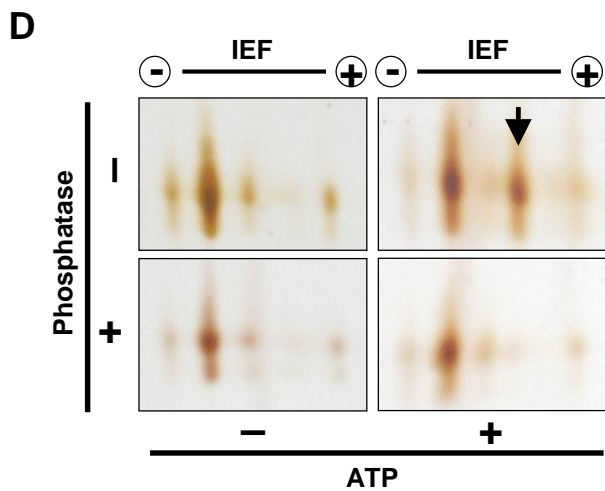

Supplement: S10 Fig — (A) After protein extraction from MCF7 cells and Syk and ezrin protein immunoprecipitation (IP), the in vitro kinase reaction is performed with [32P]-ATP either in the presence or absence of Syk inhibitor piceatannol (PIC). (B) COS7 cells are expressing FLAG-Syk (1), ezrin-GFP (2), both (4) or ezrin-GFP and FLAG-Syk kinase dead (KD) mutant (lane 3). After cell lysis and immunoprecipitation (IP) with the indicated antibodies (bottom), the in vitro kinase reaction is performed with [32P]-ATP. (C-D) COS7 cells expressing FLAG-Syk and ezrin-GFP are lysed, proteins are immunoprecipitated and the in vitro kinase reaction is performed in presence or absence of ATP. Proteins are then incubated either with alkaline phosphatase or not. Part of the reaction product is analyzed for tyrosine phosphorylation of ezrin after SDS-PAGE (C). Part of the reaction is analyzed by two-dimensional gel electrophoresis (D). Arrow designs the phosphorylated ezrin. IEF, isoelectro focusing. (PDF) [file pcbi.1005432.s017.pdf]
