# Supplementary material for: Reconstruction and signal propagation analysis of the Syk signaling network in breast cancer cells
Source: PLoS Comput Biol. 2017 Mar 17;13(3):e1005432. doi: 10.1371/journal.pcbi.1005432 (PMC5376343; doi:10.1371/journal.pcbi.1005432)
Supplement: S8 Fig — (A) MS spectrum of the cortactin heavy and light peptides containing the phosphorylated Tyr 334 residue and showing their relative abundance in pervanadate-activated MCF7 cells pretreated or not with Syk inhibitor (Pic, piceatannol). (B) MS/MS identification of the cortactin heavy peptide containing the phosphorylated Tyr334 residue. (PDF) [file pcbi.1005432.s015.pdf]

A

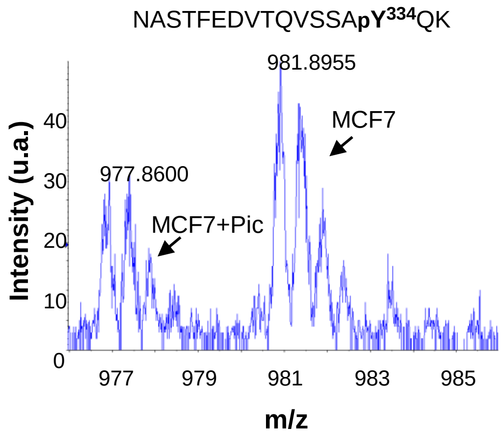

B

MS/MS Fragmentation of **NASTFEDVTQVSSApY^{334}QK**
Found in SRC8_HUMAN, **Q14247**|SRC8_HUMAN Src substrate cortactin
Homo sapiens (Human)
Match from (981.895456,2+)

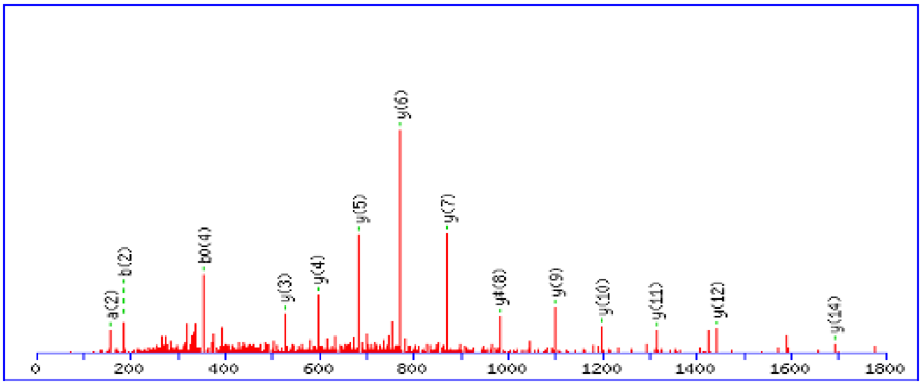

Variable modifications:
Y12 : Phospho (Y)
R20 : 13C 15N Arginine (R-full)
Ions Score: 71 Expect: 4.5e-05

#	a	a++	b	b++	b*	b*++	b0	b0++	Seq.	y	y++	y*	y*++	y0	y0++	#
1	87,06	44,03	115,05	58,03	98,02	49,52			N							17
2	158,09	79,55	186,09	93,55	169,06	85,03			A	1848,81	924,91	1831,79	916,4	1830,8	915,91	16
3	245,12	123,07	273,12	137,06	256,09	128,55	255,11	128,06	S	1777,78	889,39	1760,75	880,88	1759,77	880,39	15
4	346,17	173,59	374,17	187,59	357,14	179,07	356,16	178,58	T	1690,75	845,88	1673,72	837,36	1672,73	836,87	14
5	493,24	247,12	521,24	261,12	504,21	252,61	503,22	252,12	F	1589,7	795,35	1572,67	786,84	1571,69	786,35	13
6	622,28	311,65	650,28	325,64	633,25	317,13	632,27	316,64	E	1442,6	721,82	1425,6	713,3	1424,62	712,81	12
7	737,31	369,16	765,3	383,16	748,28	374,64	747,29	374,15	D	1313,59	657,3	1296,56	648,78	1295,58	648,29	11
8	836,38	418,69	864,37	432,69	847,35	424,18	846,36	423,69	V	1198,56	599,78	1181,53	591,27	1180,55	590,78	10
9	937,43	469,22	965,42	483,21	948,39	474,7	947,41	474,21	T	1099,5	550,25	1082,46	541,74	1081,48	541,24	9
10	1065,48	533,25	1093,48	547,24	1076,45	538,73	1075,47	538,24	Q	998,44	499,73	981,42	491,21	980,43	490,72	8
11	1164,55	582,78	1192,55	596,78	1175,52	588,26	1174,54	587,77	V	870,38	435,7	853,36	427,18	852,37	426,69	7
12	1251,59	626,3	1279,58	640,29	1262,55	631,78	1261,57	631,29	S	771,32	386,16	754,29	377,65	753,31	377,16	6
13	1338,62	669,81	1366,61	683,81	1349,59	675,3	1348,6	674,8	S	684,28	342,65	667,26	334,13	666,27	333,64	5
14	1409,65	705,33	1437,65	719,33	1420,62	710,81	1419,64	710,32	A	597,25	299,13	580,23	290,62			4
15	1652,68	826,85	1680,68	840,84	1663,65	832,33	1662,67	831,84	Y	526,22	263,61	509,19	255,1			3
16	1780,74	890,87	1808,74	904,87	1791,71	896,36	1790,73	895,87	Q	283,19	142,1	266,16	133,58			2
17									K	155,13	78,07	138,1	69,55			1
