# Supplementary material for: Reconstruction and signal propagation analysis of the Syk signaling network in breast cancer cells
Source: PLoS Comput Biol. 2017 Mar 17;13(3):e1005432. doi: 10.1371/journal.pcbi.1005432 (PMC5376343; doi:10.1371/journal.pcbi.1005432)
Supplement: S9 Fig — (A) MS spectrum of the cortactin heavy and light peptides containing the phosphorylated Tyr 446 residue and showing their relative abundance in pervanadate-activated MCF7 cells pretreated or not with Syk inhibitor piceatannol (Pic). (B) MS/MS identification of the cortactin heavy peptide containing the phosphorylated Tyr 446 residue. (PDF) [file pcbi.1005432.s016.pdf]

A

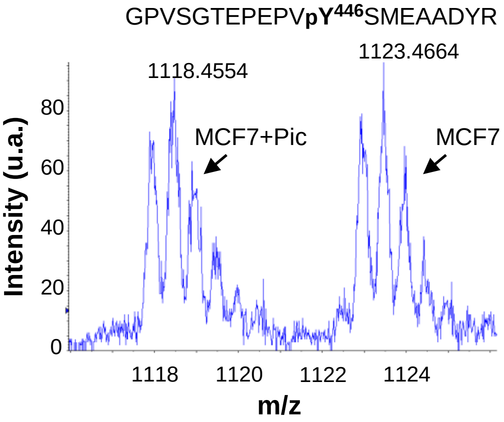

B

MS/MS Fragmentation of **GPVSGTEPEPVpY[446]SMEAADYR**
Found in SRC8_HUMAN, **Q14247**|SRC8_HUMAN Src substrate cortactin
Homo sapiens (Human)
Match from (1122.949628,2+)

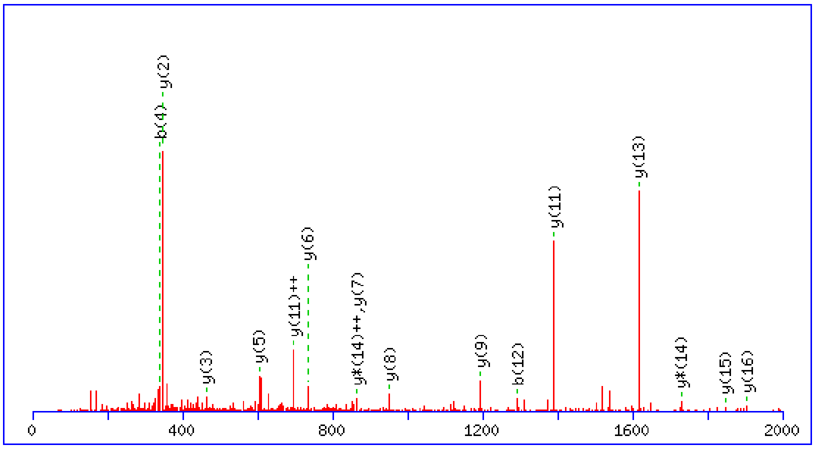

Variable modifications:
Y12 : Phospho (Y)
R20 : 13C 15N Arginine (R-full)
Ions Score: 71 Expect: 4.5e-05

#	b	b++	b*	b*++	b0	b0++	Seq.	y	y++	y*	y*++	y0	y0++	#
1	129.07	65.04	112.04	56.52			Q							16
2	200.10	100.56	183.08	92.04			A	1717.76	859.38	1700.73	850.87	1699.75	850.38	15
3	313.19	157.10	296.16	148.58			L	1646.72	823.87	1629.70	815.35	1628.71	814.86	14
4	441.25	221.13	424.22	212.61			Q	1533.64	767.32	1516.61	758.81	1515.63	758.32	13
5	556.27	278.64	539.25	270.13	538.26	269.63	D	1405.58	703.29	1388.56	694.78	1387.57	694.29	12
6	669.36	335.18	652.33	326.67	651.35	326.18	L	1290.55	645.78	1273.53	637.27	1272.54	636.78	11
7	782.44	391.72	765.41	383.21	764.43	382.72	L	1177.47	589.24	1160.44	580.73	1159.46	580.23	10
8	869.47	435.24	852.45	426.73	851.46	426.23	S	1064.39	532.70	1047.36	524.18	1046.38	523.69	9
9	998.52	499.76	981.49	491.25	980.50	490.76	E	977.35	489.18	960.33	480.67	959.34	480.18	8
10	1241.54	621.28	1224.52	612.76	1223.53	612.27	Y	848.31	424.66	831.29	416.15			7
11	1372.59	686.80	1355.56	678.28	1354.57	677.79	M	605.28	303.14	588.26	294.63			6
12	1429.61	715.31	1412.58	706.79	1411.60	706.30	G	474.24	237.62	457.22	229.11			5
13	1543.65	772.33	1526.62	763.82	1525.64	763.32	N	417.22	209.11	400.19	200.60			4
14	1614.69	807.85	1597.66	799.33	1596.68	798.84	A	303.18	152.09	286.15	143.58			3
15	1671.71	836.36	1654.68	827.84	1653.70	827.35	G	232.14	116.57	215.11	108.06			2
16							R	175.12	88.06	158.09	79.55			1
