# Supplementary material for: Reconstruction and signal propagation analysis of the Syk signaling network in breast cancer cells
Source: PLoS Comput Biol. 2017 Mar 17;13(3):e1005432. doi: 10.1371/journal.pcbi.1005432 (PMC5376343; doi:10.1371/journal.pcbi.1005432)
Supplement: S11 Fig — (PDF) [file pcbi.1005432.s018.pdf]

MS/MS Fragmentation of **SQEQLAAELAEpY^{424}TAK**
Found in EZRI_HUMAN, **P15311**|EZRI_HUMAN Ezrin
Homo sapiens (Human)
Match from (866.393092,2+)

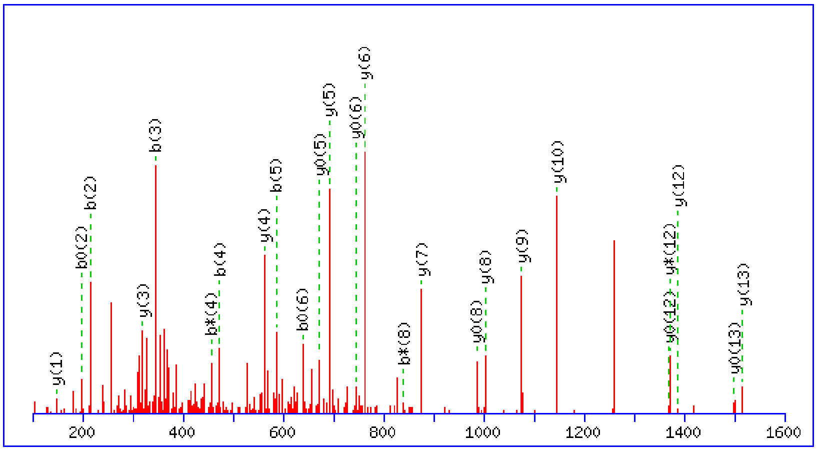

Monoisotopic mass of neutral peptide Mr(calc): 1730.78
Variable modifications:
Y12 : Phospho (Y)
Ions Score: 75 Expect: 4.1e-06
Matches (Bold Red): 25/162 fragment ions using 46 most intense peaks

#	b	b^{++}	b*	b^{*++}	b^0	b^{0++}	Seq.	y	y^{++}	y*	y^{*++}	y^0	y^{0++}	#
1	88.04	44.52			70.03	35.52	S							15
2	**216.10**	108.55	199.07	100.04	**198.09**	99.55	Q	1644.75	822.88	1627.73	814.37	1626.74	813.87	14
3	**345.14**	173.07	328.11	164.56	327.13	164.07	E	**1516.69**	758.85	1499.67	750.34	**1498.68**	749.84	13
4	**473.20**	237.10	**456.17**	228.59	455.19	228.10	Q	**1387.65**	694.33	**1370.62**	685.82	**1369.64**	685.32	12
5	**586.28**	293.65	569.26	285.13	568.27	284.64	L	1259.59	630.30	1242.57	621.79	1241.58	621.29	11
6	657.32	329.16	640.29	320.65	**639.31**	320.16	A	**1146.51**	573.76	1129.48	565.24	1128.50	564.75	10
7	728.36	364.68	711.33	356.17	710.35	355.68	A	**1075.47**	538.24	1058.44	529.73	1057.46	529.23	9
8	857.40	429.20	**840.37**	420.69	839.39	420.20	E	**1004.43**	502.72	987.41	494.21	**986.42**	493.72	8
9	970.48	485.75	953.46	477.23	952.47	476.74	L	**875.39**	438.20	858.36	429.69	857.38	429.19	7
10	1041.52	521.26	1024.49	512.75	1023.51	512.26	A	**762.31**	381.66	745.28	373.14	**744.30**	372.65	6
11	1170.56	585.79	1153.54	577.27	1152.55	576.78	E	**691.27**	346.14	674.24	337.63	**673.26**	337.13	5
12	1413.59	707.30	1396.57	698.79	1395.58	698.30	Y	**562.23**	281.62	545.20	273.10	544.22	272.61	4
13	1514.64	757.82	1497.61	749.31	1496.63	748.82	T	**319.20**	160.10	302.17	151.59	301.19	151.10	3
14	1585.68	793.34	1568.65	784.83	1567.67	784.34	A	218.15	109.58	201.12	101.07			2
15							K	**147.11**	74.06	130.09	65.55			1
