# Supplementary material for: Reconstruction and signal propagation analysis of the Syk signaling network in breast cancer cells
Source: PLoS Comput Biol. 2017 Mar 17;13(3):e1005432. doi: 10.1371/journal.pcbi.1005432 (PMC5376343; doi:10.1371/journal.pcbi.1005432)
Supplement: S1 File — Data representing the full Syk network and the subnetworks analyzed in the paper. An example of web visualization of the Syk networks based on a customized Cytoscape Web. (ZIP) [file pcbi.1005432.s019.zip › networks&code/webnet/cortactin.html]

cytoscape-js demo


# cytoscape-js demo

Select a node to see more
